# Lead and calcium crosstalk tempted acrosome damage and hyperpolarization of spermatozoa: signaling and ultra-structural evidences

**DOI:** 10.1186/s40659-024-00517-x

**Published:** 2024-07-05

**Authors:** Rajkumar Singh Yadav, Bhawna Kushawaha, Rahul Dhariya, Dilip Kumar Swain, Brijesh Yadav, Mukul Anand, Priyambada Kumari, Pradeep Kumar Rai, Dipty Singh, Sarvajeet Yadav, Satish Kumar Garg

**Affiliations:** 1 Department of Pharmacology and Toxicology, U.P. Pandit Deen Dayal Upadhyaya Pashu Chikitsa Vigyan Vishwavidyalaya Evam Go Anusandhan Sansthan, Mathura, India; 2 College of Biotechnology, Mathura, India; 3 Department of Veterinary Physiology, U.P. Pandit Deen Dayal Upadhyaya Pashu Chikitsa Vigyan Vishwavidyalaya Evam Go Anusandhan Sansthan, Mathura, India; 4 BD Bioscience, Gurgaon, India; 5 ICMR-National Institute for Research in Reproductive Health (NIRRH), Mumbai, India; 6 U.P. Pandit Deen Dayal Upadhayaya Pashu Chikitsa Vigyan Vishwavidyalaya Evam Go Anusandhan Sansthan (DUVASU), Mathura, Uttar Pradesh 281001 India; 7 Present Address: University of Nebraska Medical Center (UNMC), Omaha, USA

**Keywords:** Spermatozoa, Lead, Ca^2+^ channels, DNA fragmentation, Capacitation and electron microscopy

## Abstract

**Background:**

Exposure of humans and animals to heavy metals is increasing day-by-day; thus, lead even today remains of significant public health concern. According to CDC, blood lead reference value (BLRV) ranges from 3.5 µg/dl to 5 μg/dl in adults. Recently, almost 2.6% decline in male fertility per year has been reported but the cause is not well established. Lead (Pb^2+^) affects the size of testis, semen quality, and secretory functions of prostate. But the molecular mechanism(s) of lead toxicity in sperm cells is not clear. Thus, present study was undertaken to evaluate the adverse effects of lead acetate at environmentally relevant exposure levels (0.5, 5, 10 and 20 ppm) on functional and molecular dynamics of spermatozoa of bucks following in vitro exposure for 15 min and 3 h.

**Results:**

Lead significantly decreased motility, viable count, and motion kinematic patterns of spermatozoa like curvilinear velocity, straight-line velocity, average path velocity, beat cross frequency and maximum amplitude of head lateral displacement even at 5 ppm concentration. Pb^2+^ modulated intracellular cAMP and Ca^2+^ levels in sperm cells through L-type calcium channels and induced spontaneous or premature acrosome reaction (AR) by increasing tyrosine phosphorylation of sperm proteins and downregulated mitochondrial transmembrane potential. Lead significantly increased DNA damage and apoptosis as well. Electron microscopy studies revealed Pb^2+^ -induced deleterious effects on plasma membrane of head and acrosome including collapsed cristae in mitochondria.

**Conclusions:**

Pb^2+^ not only mimics Ca^2+^ but also affects cellular targets involved in generation of cAMP, mitochondrial transmembrane potential, and ionic exchange. Lead seems to interact with Ca^2+^ channels because of charge similarity and probably enters the sperm cell through these channels and results in hyperpolarization. Our findings also indicate lead-induced TP and intracellular Ca^2+^ release in spermatozoa which in turn may be responsible for premature acrosome exocytosis which is essential feature of capacitation for fertilization. Thus, lead seems to reduce the fertilizing capacity of spermatozoa even at 0.5 ppm concentrations.

**Graphical Abstract:**

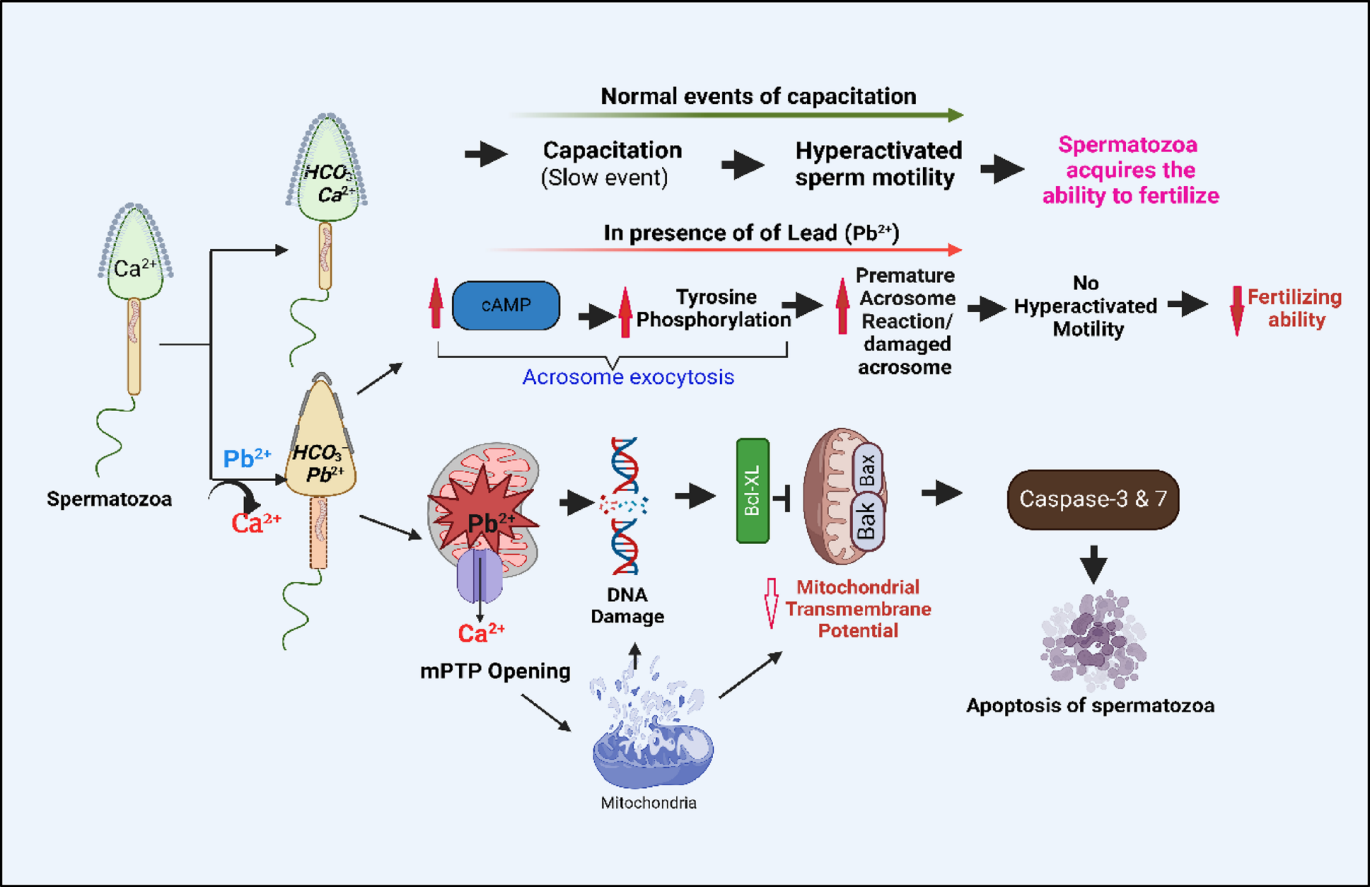

## Background

World Health Organization’s [1] report states that nearly half of the two million lives were lost due to lead exposure in 2019. Primary sources of lead exposure are soil, water, toys, batteries, cosmetics, food, paint, and industrial waste. As per the review article by Levine et al. [2], on an average male fertility is declining by 2.6% per annum. There could be several reasons for the same, but pollution seems to be the major player. Heavy metals are most persistent environmental pollutants as these are barely biodegraded once introduced into environment. Heavy metals (mercury-Hg, cadmium-Cd and copper-Cu) have been reported to significantly decrease progressive motility and viable count of exposed spermatozoa [3, 4]. These heavy metals are known to affect the size of testis, semen quality, secretory functions of prostate and seminal vesicles, and reproductive endocrine functions which ultimately result in loss of libido and fertility and/or result in impotence [5–7]. Presence of heavy metals in seminal fluid [8, 9] suggests that functional attributes of sperm cells including their competence for successful fertilization is likely to be adversely affected by heavy metals.

Capacitation of sperm cells, involving an increase in membrane fluidity, cholesterol efflux and ion fluxes, is an important phenomenon for successful fertilization which results in alteration of sperm membrane potential, increases tyrosine phosphorylation of proteins, and induces hyper-activation and acrosome reaction [10]. Intracellular cAMP induces phosphorylation of the axonemal dynein by protein kinase A (PKA) [11, 12] to increase flagella beating and sperm motility [13]. In sperms, Ca^2+^ is associated with significant functions like sperm motility, capacitation, chemotaxis and acrosome exocytosis [14]. To successfully navigate the female reproductive tract, spermatozoon senses the environment and accordingly adjusts its motility which is controlled by ATP production and flagellar ion-homeostasis. Swimming behavior of spermatozoa is controlled by rise in [Ca^2+^]_i_ that changes flagellar beat pattern through Ca^2+^ sensing proteins calaxins [15, 16]. Voltage-dependent Ca^2+^ channels (VOCCs) play an important role in ion influx into sperms [15]. Localization of various VOCCs in head and flagellum of sperm cells suggest that these are involved in modulation of sperm motility and acrosome reaction (AR), and both these important events require Ca^2+^ [17, 18].

Calcium bound calaxins inhibit activity of dynein motors within axoneme of the cell and result in high-amplitude asymmetric bending of flagella [19]. Exposure to high levels of cadmium [20] and lead [21] is reported to increase reactive oxygen species (ROS) production in testes and decrease sperms production, antioxidant capacity, and motility [22]. Cadmium on in-vitro exposure is reported to induce apoptosis in human sperm cells due to increase in caspase activity [23] while mercury induced necrosis in goat spermatozoa [24], thus, suggesting heavy metals trigger apoptosis or necrosis pathways. Despite plethora of literature on toxic effects of heavy metals on testes and sperm cells, not much information is available on how heavy metals, especially lead modulates the functional attributes of sperms. Thus, the objective of present study was to determine the effects of different concentrations of lead acetate on kinematic patterns of sperm and molecular signaling mechanism(s) of sperm functions including capacitation. Our results indicate that lead significantly decreased motility and altered motion kinematic patterns of spermatozoa even at 0.5 ppm lead concentration. Further, Pb^2+^ modulated intracellular cAMP and Ca^2+^ levels in sperm cells and induced premature acrosome reaction (AR) by tyrosine phosphorylation of sperm proteins and significantly down-regulated mitochondrial trans-membrane potential and resulted in DNA damage and apoptosis.

## Results

**Fig. 1:**
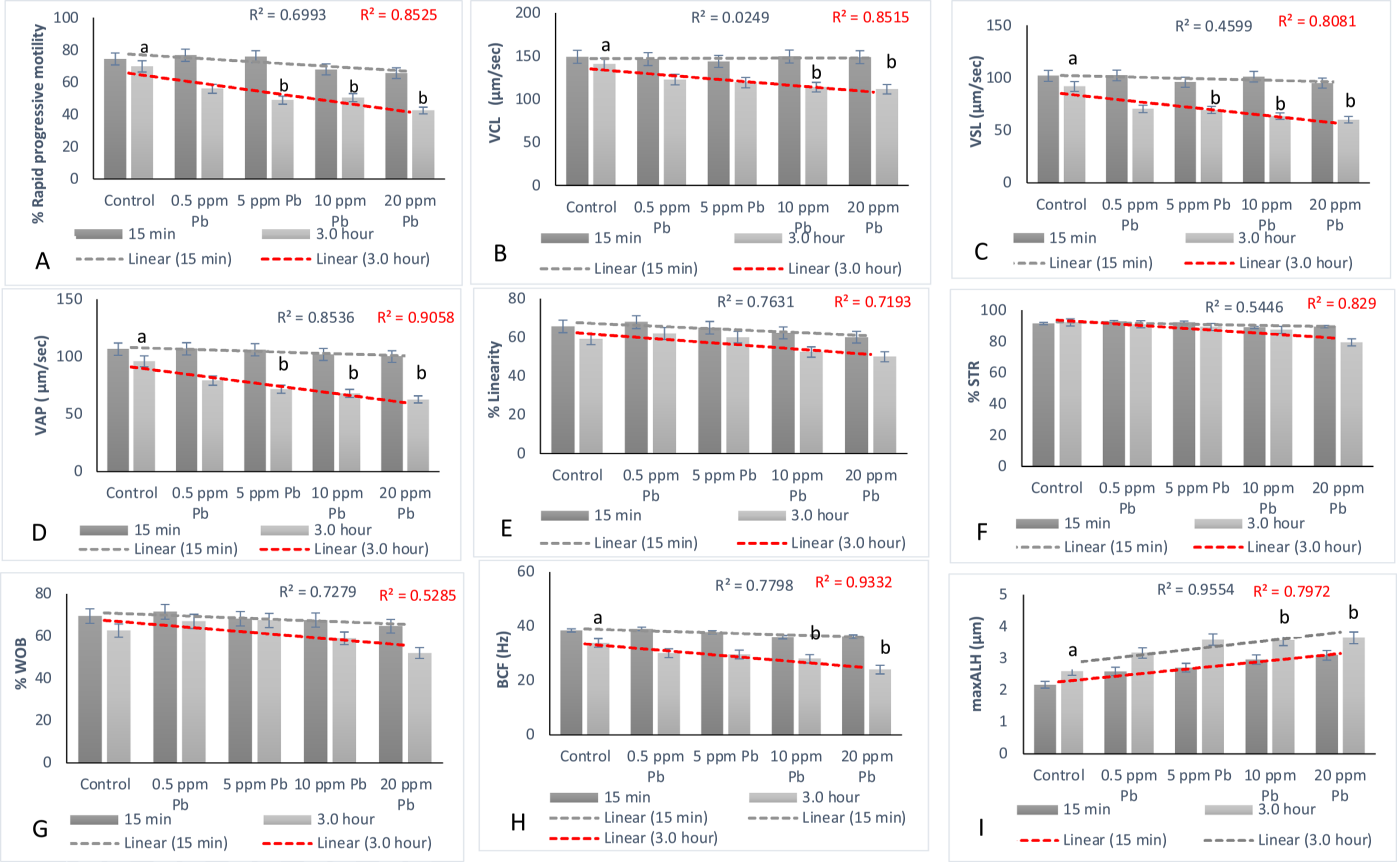
**A** Progressive motility percent, and other motion kinematic patterns of spermatozoa like **B** curvilinear velocity (VCL; µm s^−1^), **C** straight-line velocity (VSL; µm s^−1^), **D** average path velocity (VAP; µm s^−1^), **E** linearity (LIN; %), **F** straightness (STR; %), **G** wobble (WOB; %), **H** beat cross frequency (BCF; Hz), and **I** maximum amplitude of head lateral displacement (maxALH; µm) of spermatozoa following in-vitro exposure to different concentrations of lead acetate (0.5–20 ppm) at 15 min and 3 h. Data presented are mean ± SE of the semen samples of six bucks. Vertical bars represent the standard error (SE). Different superscripts on bars indicate significant (p < 0.05) differences

**Fig. 2:**
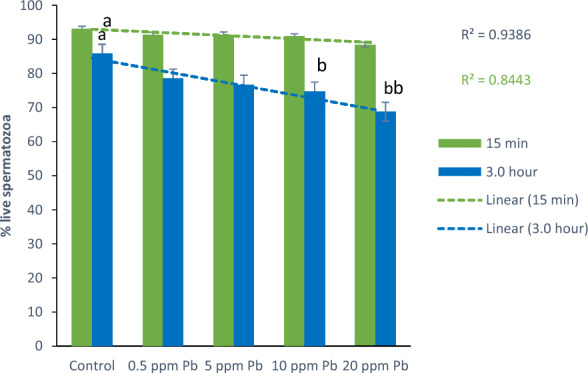
Total live percent of sperm cells following *in-vitro* exposure of semen samples to different concentrations of lead acetate (0.5–20 ppm) at different time intervals. Data presented are mean ± SE of the semen samples of six bucks. Vertical bars represent the standard error (SE). Different superscripts on bars indicate significant (p < 0.05) differences

**Fig. 3:**
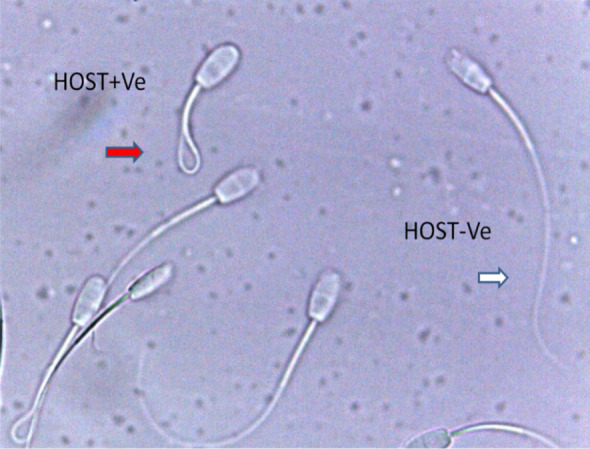
Percent HOS-positive spermatozoa at different time intervals following in-vitro exposure of semen samples to different concentrations of lead acetate (0.5–20 ppm). Spermatozoa showing HOS-positive response (curled tail) and HOS-negative response (no curling of tail)

**Fig. 4:**
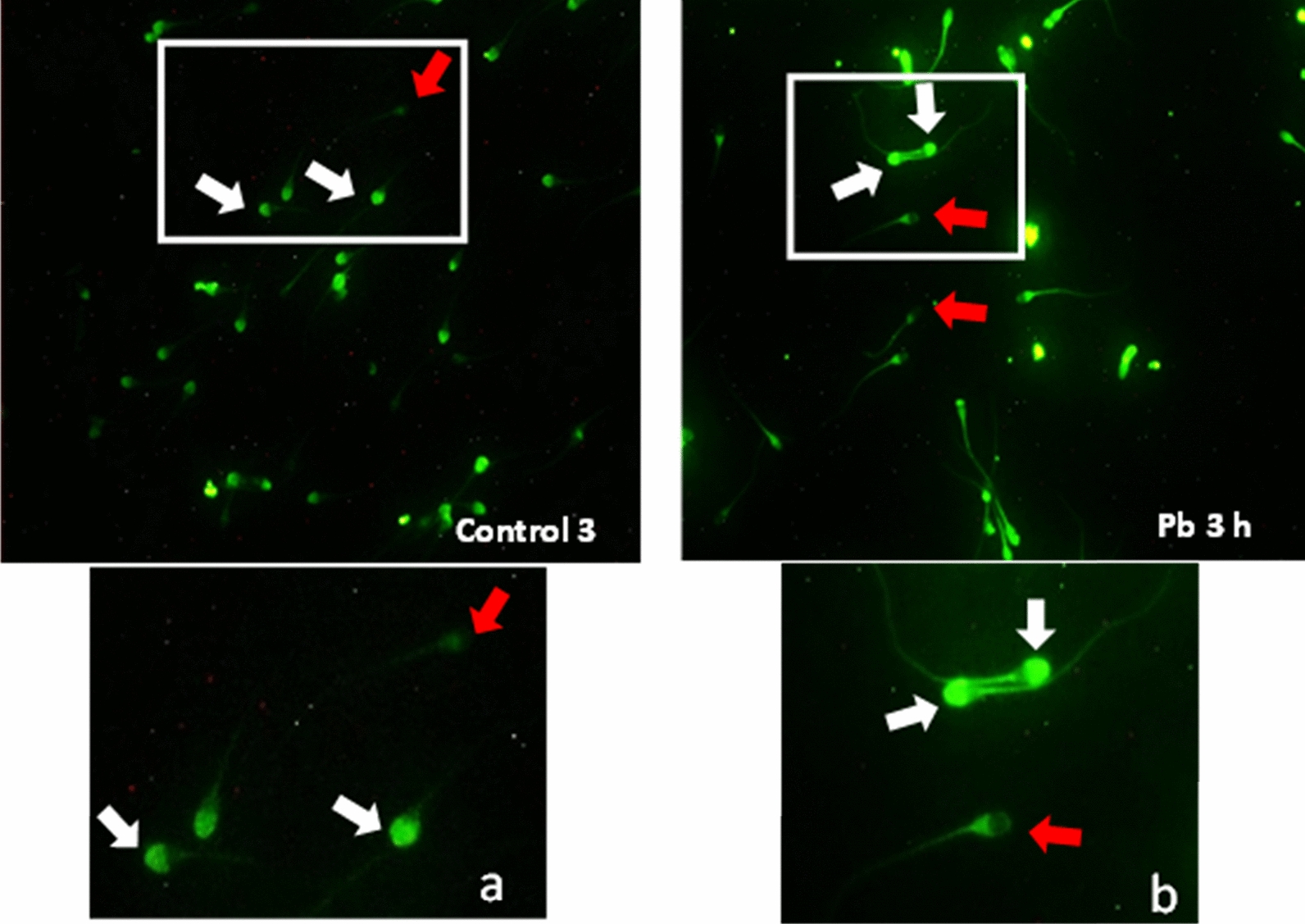
Acrosome status in sperm cells of the control (**a**; zoom image) and lead acetate (**b**; zoom image) treated groups after 3 h of incubation. White arrows indicate the sperm cells with intact acrosome while the red arrows show the sperm cells having damaged acrosome (loss of acrosome) (40 ×)

**Fig. 5:**
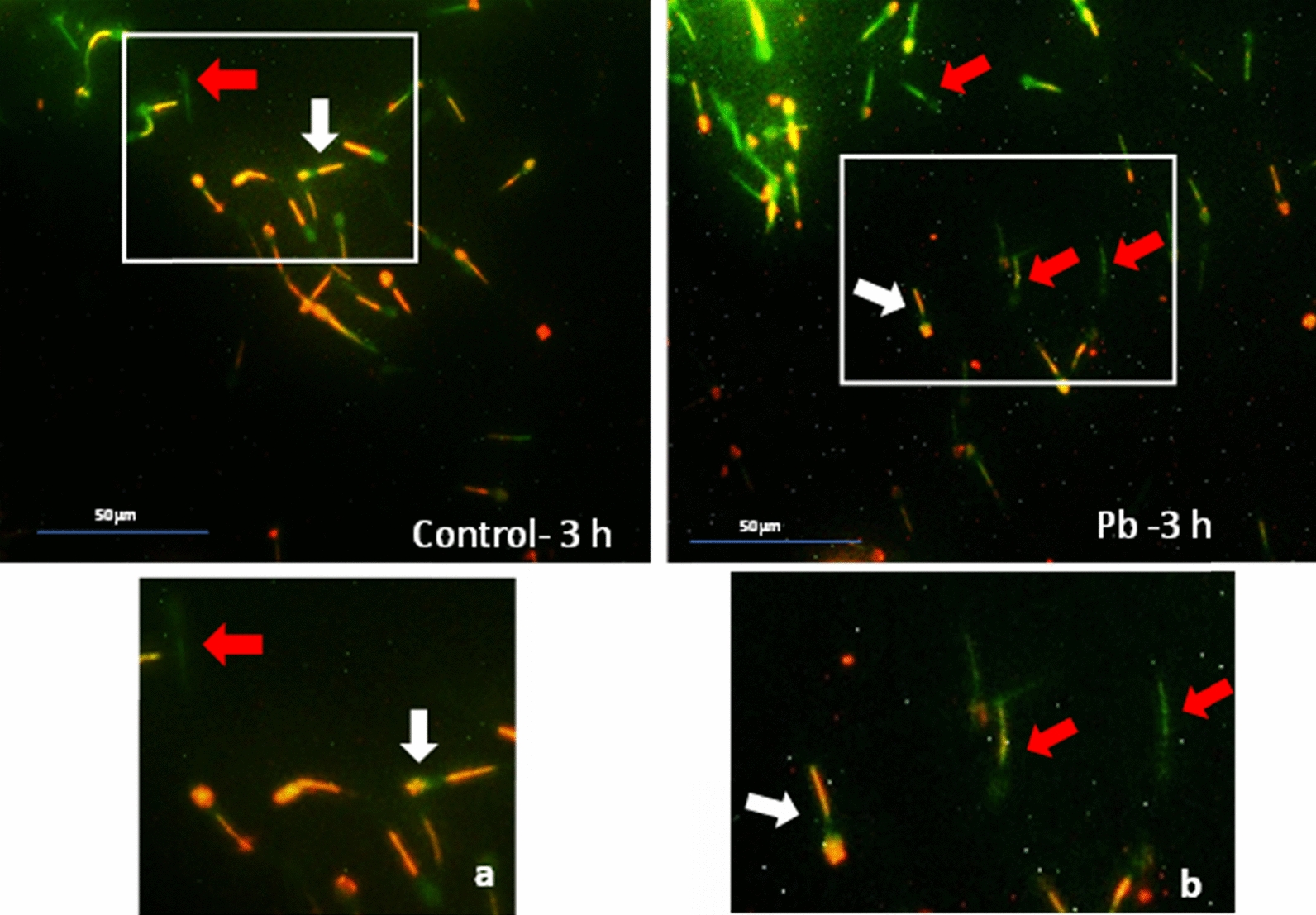
Mitochondrial transmembrane potential (MTP) of sperm cells in the control (**a**; zoom image) and lead acetate (**b**; zoom image) treated groups after 3 h of incubation. White arrows mark the spermatozoa with high MTP while red arrows mark the spermatozoa with low MTP (40 ×)

**Fig. 6:**
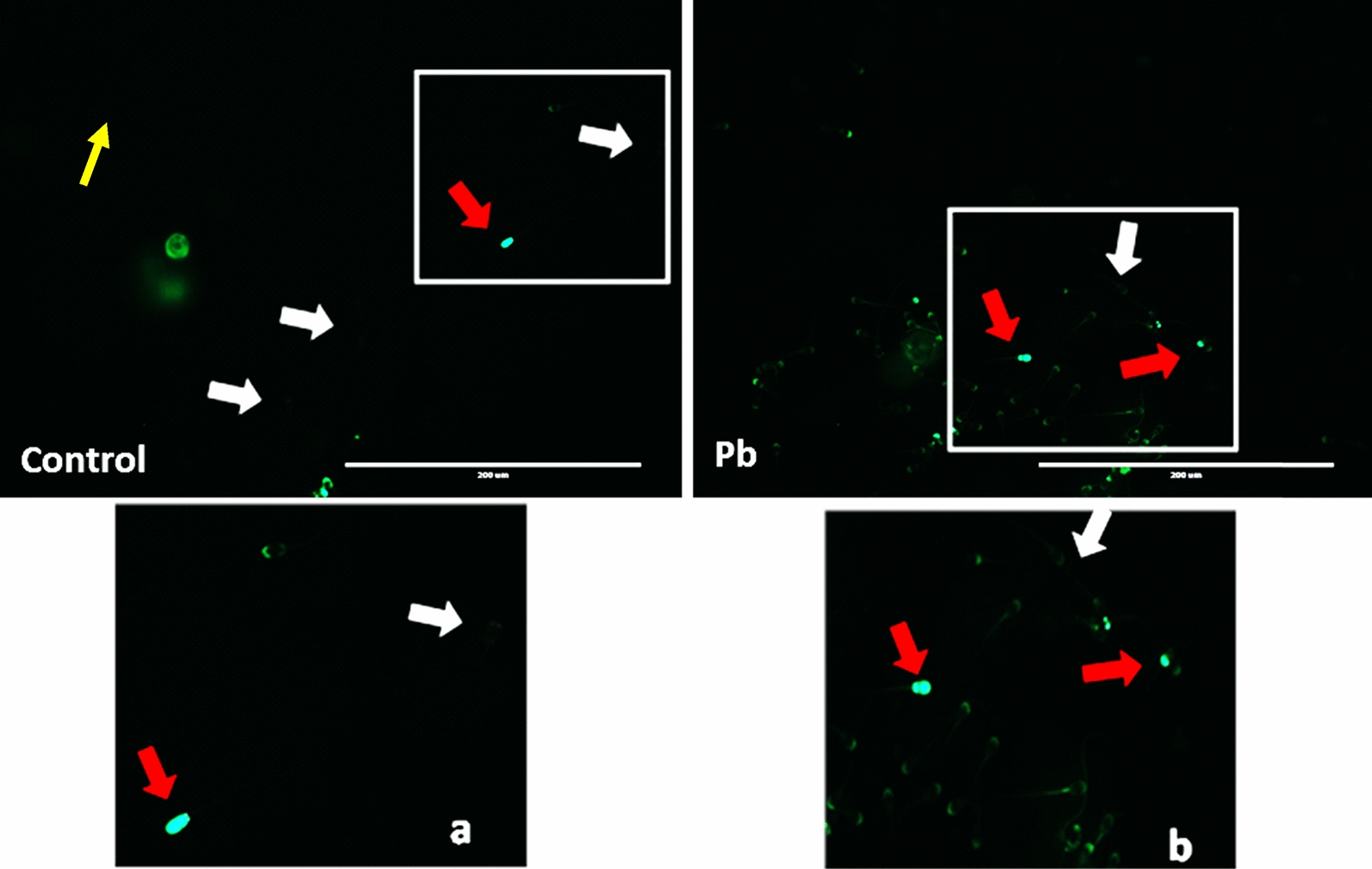
Buck spermatozoa of the Control (**a**; zoom image) and lead acetate (**b**; zoom image) treated groups showing TUNEL positive and TUNEL negative responses after 3 h. Red arrows mark the spermatozoa with TUNEL positive (DNA damage) while the white arrows mark at sperm cells showing TUNEL negative response i.e. no DNA damage (20 ×)

**Fig. 7:**
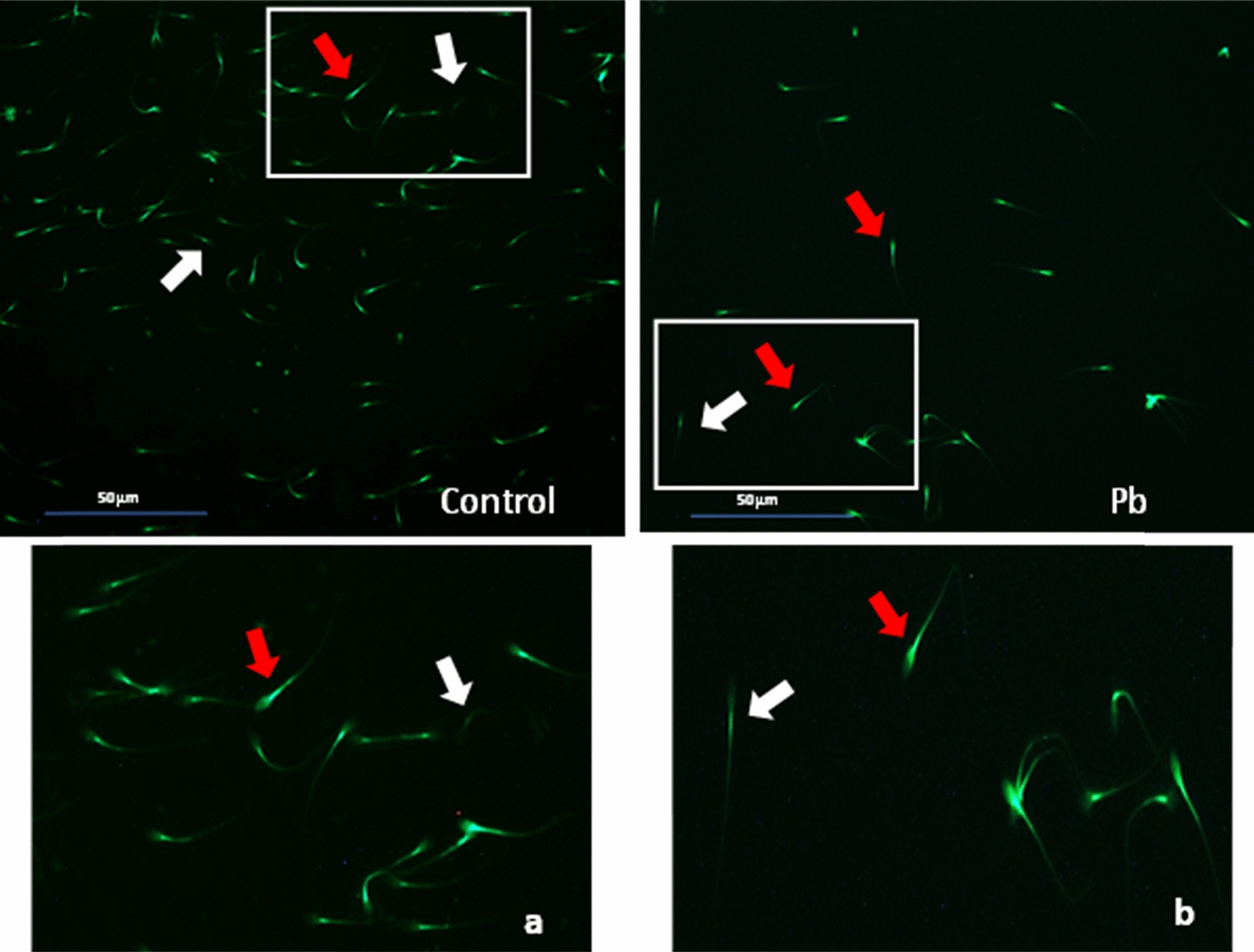
Representative images of caspase 3 and 7 activities in sperm cells of the control (**a**; zoom image) and lead acetate (**b**; zoom image) treated groups after 3 h of incubation. Caspase 3 and 7 negative spermatozoa are marked with white arrow while Caspase 3 and 7 positive spermatozoa are marked with red arrow (20 ×)

**Fig. 8:**
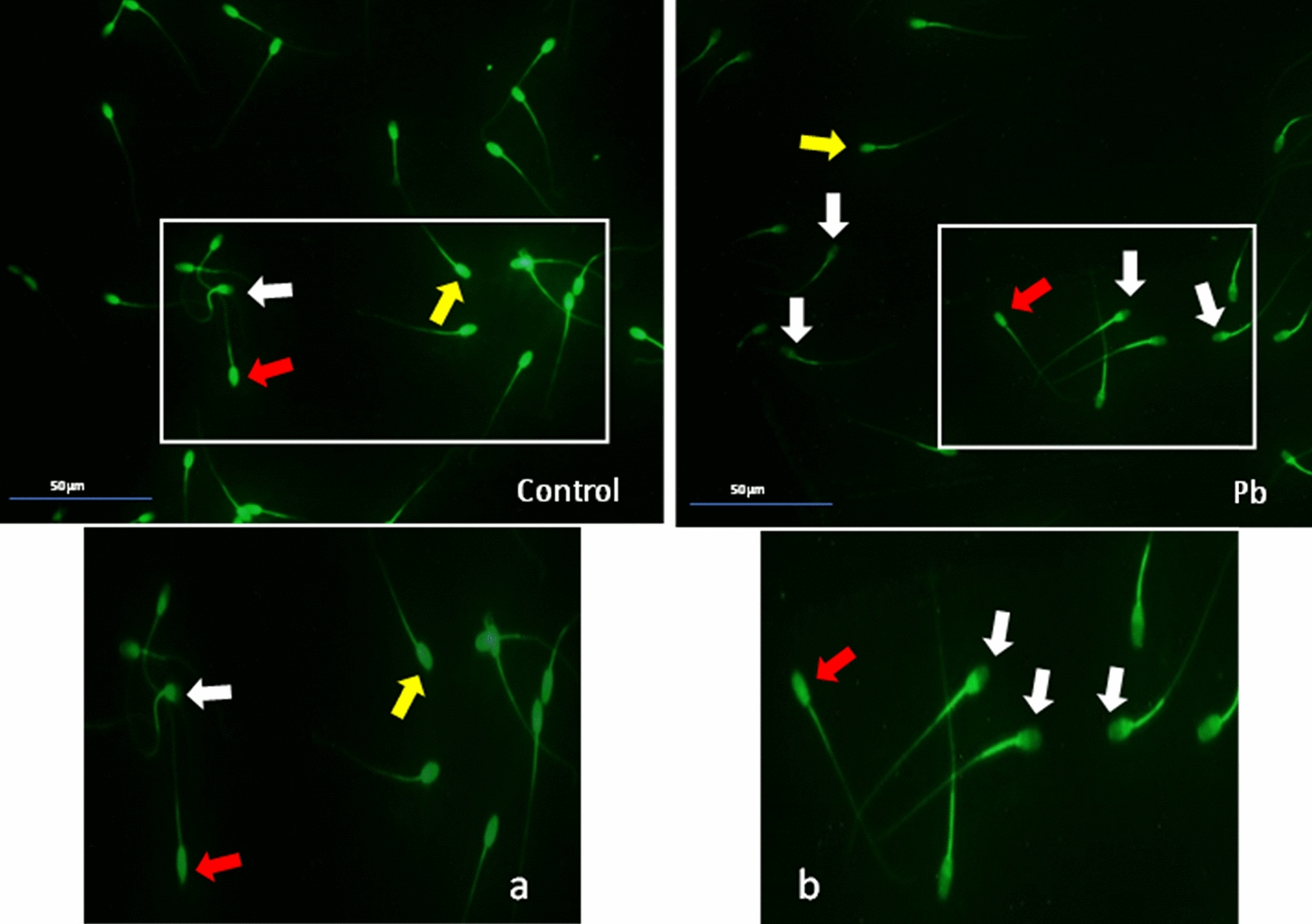
Buck spermatozoa of the Control (**a**; zoom image) and lead acetate treated (**b**; zoom image) groups after 3 h of incubation showing capacitation status. Arrows indicate different types of patterns of capacitation in sperm cells (F Pattern-Yellow arrow, B Pattern-Red arrow, and AR Pattern-White arrow) at 40 ×.

**Fig. 9:**
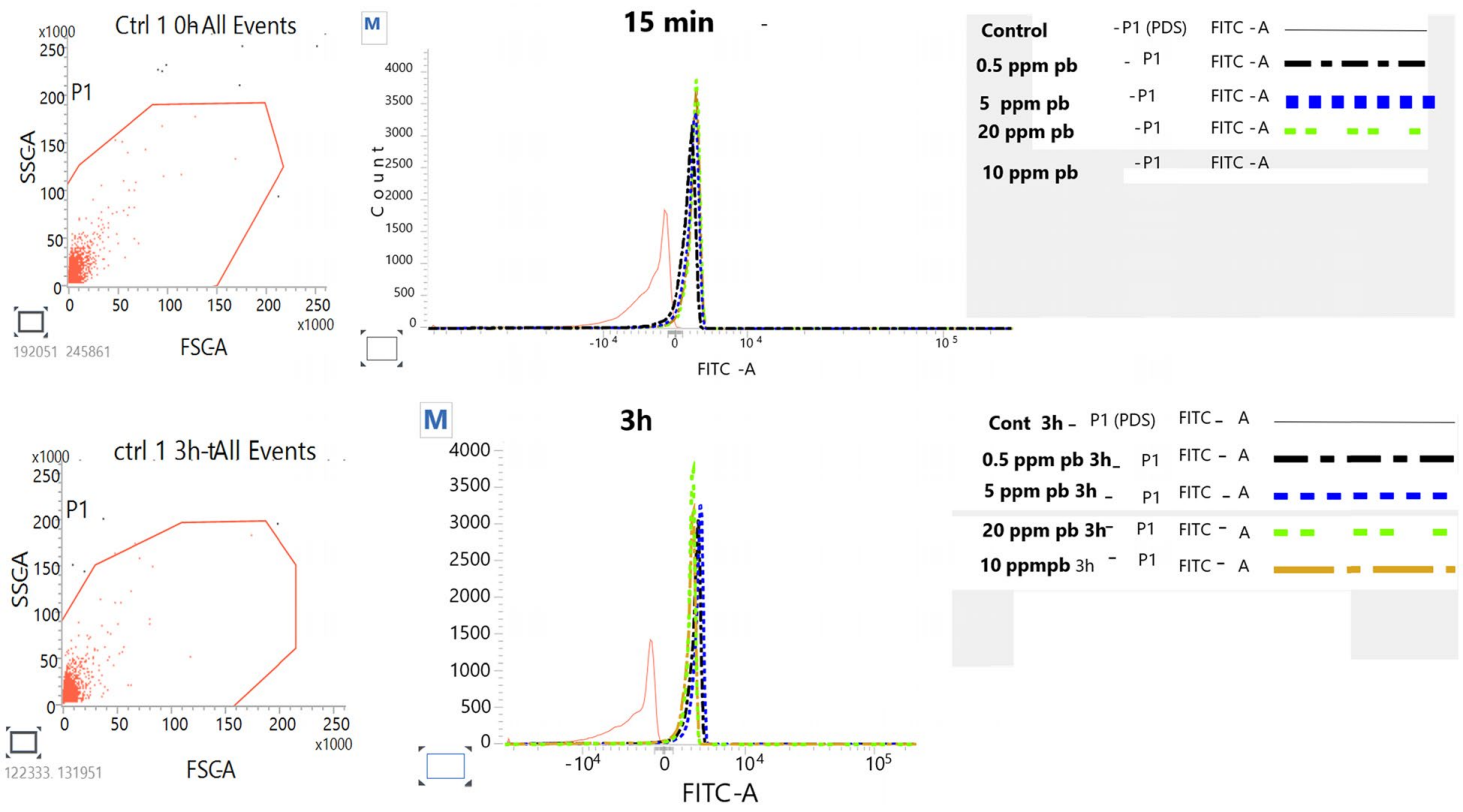
Overlay of intracellular Ca^+2^ release in percent sperm cells following in vitro exposure to different concentrations of lead acetate after 15 min and 3 h

**Fig. 10:**
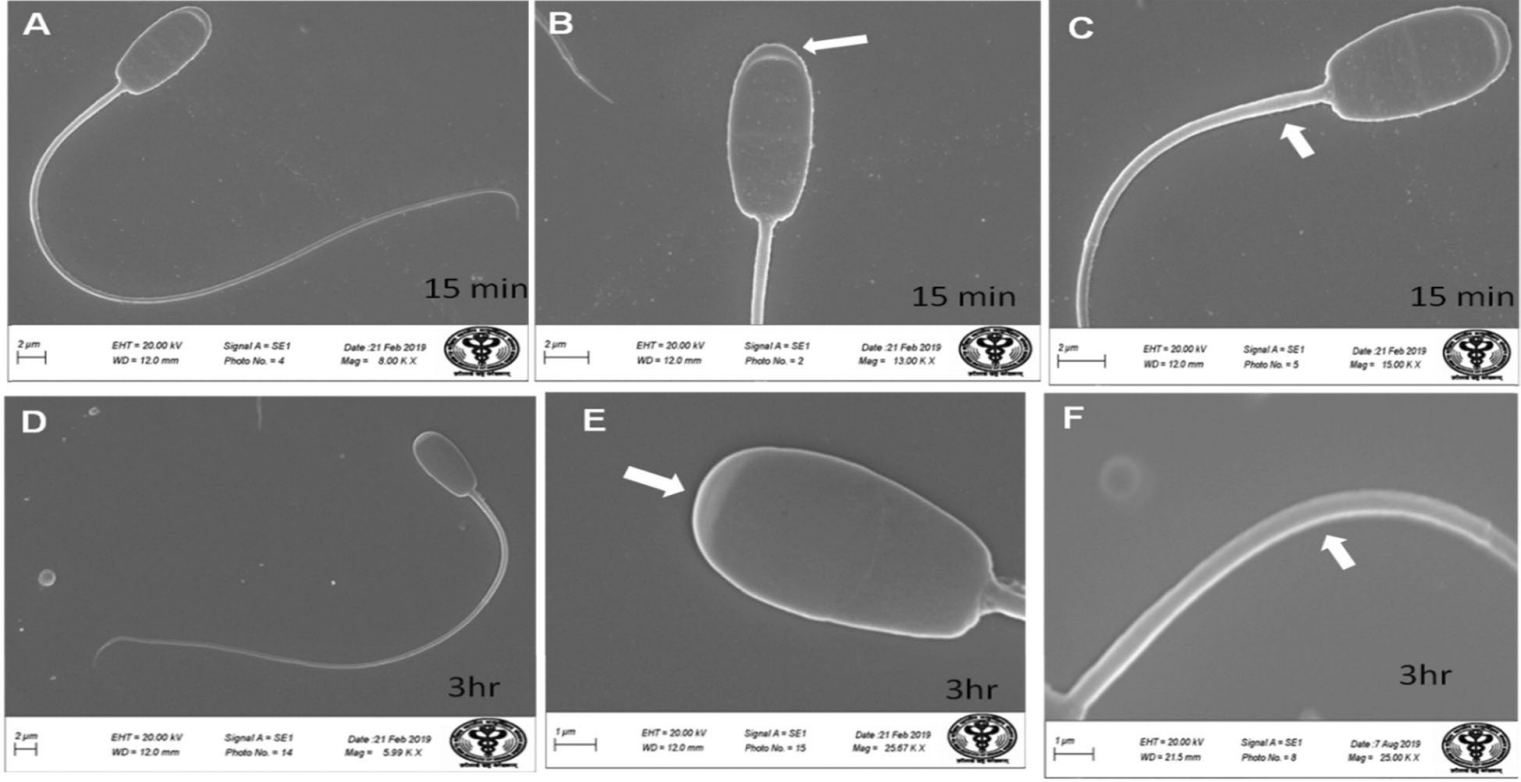
Scanning electron microscopic images of buck spermatozoa of the control group showing intact surface (**A**, **D**), intact acrosome (**B**, **E**), middle piece (**C** and **F**) and neck and tail (**D**) after 15 min and 3 h

**Fig. 11:**
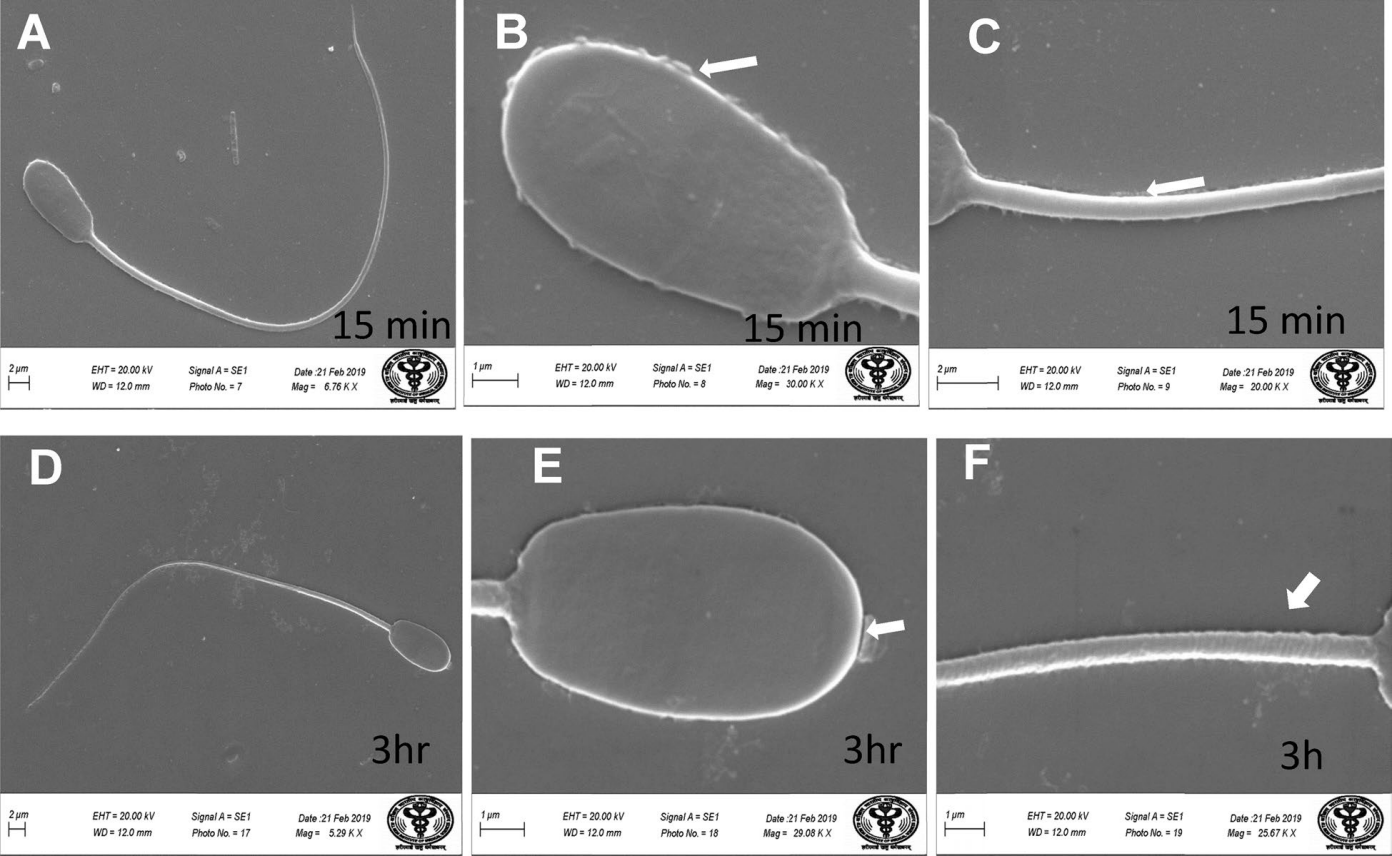
Scanning Electron Microscopy images of the buck spermatozoa following in vitro exposure to 5 ppm lead acetate for 15 min and 3 h showing intact surface structure of sperm cell (**A**) deformities in head (**B**) and middle piece (**C** and **D**), swollen acrosome (**E**) and wavy middle piece (**F**)

**Fig. 12:**
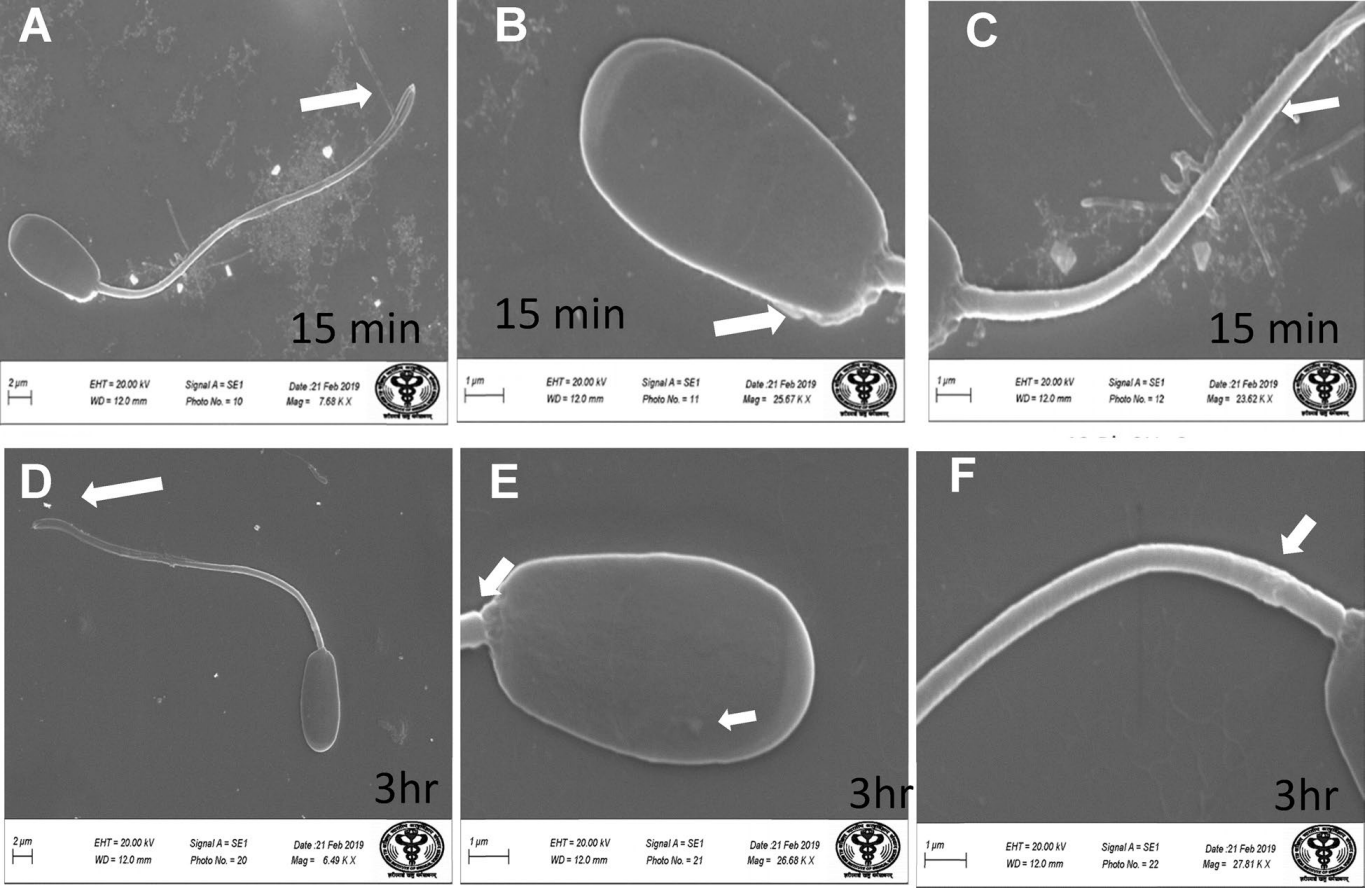
Scanning Electron Microscopy images of the buck spermatozoa following in vitro exposure to 20 ppm lead acetate for 15 min and 3 h showing swollen tail and plasma membrane (**A** and **D**), disturbed membrane integrity of the sperm head (**B** and **E**), swollen mid piece (**C**) and bent neck (**E** and **F**)

1. *Effect of lead on sperm cells motility*: Data presented in Fig. 1A–I shows that compared to the control (70%), there was significant (p < 0.05) decrease in progressive motility up to 48, 50 and 42% following exposure to different concentrations of lead acetate at 5, 10, and 20 ppm, respectively, for 3 h. Compared to the control group, different velocities of spermatozoa like VCL, VSL, VAP (µm^s−1^), and BCF (hyperactivation) were also found to be significantly decreased after 3 h exposure to lead, but maxALH (µm) was found to be increased at higher concentration of lead which altogether indicated sluggish movement of sperm cells.
2. *Effect of lead on sperm cells viability*: Data illustrated in Fig. 2 shows that compared to control, there was no change in percentage of the viable sperm cells following *in-vitro* exposure to different concentrations of lead acetate (0.5, 5, 10, and 20 ppm) for 15 min. But compared to the control (85.8%) group, significant decrease in viable spermatozoa (68.68%) was observed in 20 ppm lead acetate treatment group after 3 h which indicates concentration- and time-dependent adverse effect of lead acetate on sperm viability.
3. *Effect of lead on membrane integrity of sperm cells*: Data summarized in Fig. 3 illustrates the effect of in-vitro exposure of buck spermatozoa to lead acetate for 15 min and 3 h on membrane integrity of sperm cells which is expressed as percentage of HOS-positive sperm cells. Perusal of the data revealed that compared to control, there was no significant (p > 0.05) decrease in HOS-positive sperm cells after 15 min of exposure to different concentrations of lead acetate (0.5, 5, 10 and 20 ppm). However, HOS- positive sperm cells percentage differed significantly after 3 h of exposure to 20 ppm lead (48.66%) compared to 64.31% in control group. Therefore, our data evidently suggests that lead acetate affected membrane integrity of spermatozoa after exposure to higher concentration of lead.
4. *Effect of lead on acrosome integrity*: Perusal of the data shown in Table 1 illustrates the effect of lead acetate on acrosomal integrity of buck sperm cells following in-vitro exposure to different concentrations of lead (0.5, 5, 10 and 20 ppm) at different time intervals. Following exposure to 0.5, 5, 10 and 20 ppm concentrations of lead acetate for 15 min, FITC-PSA positive spermatozoa (with intact acrosome) did not significantly differ from those in the control group (Fig. 4). However, after 3 h, 66.68, 63.81, 58.50 and 47.01% sperms were found to have intact acrosome in 0.5, 5, 10 and 20 ppm lead acetate treated group, respectively. Sperms of highest lead acetate concentration group (20 ppm) after 3 h showed significant (p < 0.05) decrease in percentage of sperm cells with intact acrosome (47.01%) compared to the control group (68.83%). Further, significant decrease in per cent sperm cells with intact acrosome were observed in all the lead acetate treated groups (0.5, 5, 10 and 20 ppm) after 3 h compared to the corresponding values in 15 min exposure groups. Therefore, our data suggest that acrosomal integrity of sperm cells was not only affected by increase in concentration of lead but also by exposure time i.e. on prolonged exposure there was more pronounced effect on acrosomal integrity.
5. *Effect of lead on mitochondrial trans-membrane potential (MTP)*: Data presented in Table 1 and illustrated in Fig. 5 shows the effect of different concentrations of lead acetate (0.5, 5, 10 and 20 ppm) on mitochondrial trans-membrane potential of sperm cells as determined by JC-1 staining at 15 min and 3 h post-exposure periods and expressed as percent (%) spermatozoa showing MTP response. Perusal of the data in Table 1 revealed that compared to control group, there was no significant (p > 0.05) difference in percentage of sperm cells showing MTP in 0.5, 5 and 10 ppm lead acetate exposed groups after 15 min. However, 20 ppm lead acetate resulted in significant (p < 0.05) decrease in sperm cells showing MTP compared to the control group after 15 min of exposure. Similarly, compared to the control group, there was no significant (p > 0.05) difference in sperm cells showing mitochondrial trans-membrane potential in lead acetate treated groups (0.5, 5, and 10 ppm) after 3 h. But significant (p < 0.05) decrease in number of sperm cells (51.13%) showing mitochondrial transmembrane potential was observed in 20 ppm lead acetate treated group after 3 h compared to the control group (82.42%).
6. *Effect of lead on DNA damage*: Perusal of the data summarized in Table 1 and illustrated in Fig. 6 revealed that there was significant (p < 0.05) increase in percentage count of TUNEL positive spermatozoa (Fig. 6) in 0.5, 5, 10 and 20 ppm lead acetate treated groups after15 min exposure compared to the control group. Even the lowest used concentration of lead acetate (0.5 ppm) produced significant (p < 0.05) damage to spermatozoa DNA within 15 min of exposure. After 15 min, 7.84% of sperm cells showed DNA damage in 20 ppm lead acetate-treated group and this value was significantly (p > 0.05) higher compared to the values observed in control and 0.5, 5 and 10 ppm lead acetate-treated groups. When sperm cells were exposed to same concentrations (0.5, 5, 10 and 20 ppm) of lead for 3 h, significantly higher damage to DNA of sperm cells was observed even at the lowest used concentration (0.5 ppm) of lead acetate. In the highest used dose group of lead acetate (20 ppm), DNA damage was found to be in 15.79% sperm cells compared to just 4.02% in control group. Compared to the exposure to lead acetate (0.5, 5, 10 and 20 ppm) for 15 min, significant (p < 0.05) increase in % sperm cells showing DNA damage was observed after 3 h. Thus, suggesting that lead acetate produced concentration as well as time-dependent damage in buck spermatozoa DNA.
7. *Effect of lead on Caspase 3 and 7 activities*: Perusal of the data summarized in Table 1 and shown in Fig. 7 revealed that after 15 min, compared to the control and 0.5 ppm lead acetate-treated groups, percentage of caspase 3 and 7 positive spermatozoa (Fig. 7) were found to be significantly (p < 0.05) higher in 5, 10 and 20 ppm lead acetate treated groups. Percentage of caspase 3 and 7 positive sperm cells was almost double (33.76%) in 20 ppm lead acetate treatment group compared to just 16.44% in control group. After 3 h also, caspase 3 and 7 positive sperm cells percentage were found to be increased in concentration-dependent manner as the values were significantly (p < 0.05) higher in 5, 10 and 20 ppm lead acetate treated groups compared to the control and 0.5 ppm lead acetate treated groups, However, caspase 3 and 7 positive spermatozoa percentages did not significantly (p > 0.05) differ between different times of exposure (15 min and 3 h) in all lead treated groups. Thus, our data evidently suggest that effect of lead acetate (5–20 ppm) on caspase 3 and 7 activity was concentration-dependent both at 15 min and 3 h. However, exposure time apparently did not have any significant effect on caspase 3 and 7 activity as the values at 15 min and 3 h did not significantly differ from each other.
8. *Effect of lead on capacitation like changes*: Data summarized in Table 2 and shown in Fig. 8 show the effect of different concentrations of lead acetate (0.5–20 ppm) on capacitation like changes (F, B and AR- patterns) in sperm cells, as determined by CTC staining method, following exposure for 15 min and 3 h. Perusal of the data revealed that compared to control group (94.59%), there was no significant difference in percentage of sperm cells showing F, B and AR patterns following exposure to lead acetate (0.5–20 ppm) for 15 min. However, compared to control (83.27%), F pattern significantly decreased (54.66%) in 20 ppm lead acetate treated group after 3 of exposure. Although count of sperm cells showing B-pattern after 3 h exposure to lead increased in concentration dependent manner, but the difference was not statistically significant just like after 15 min of exposure. However, there was significant (p < 0.05) increase in percentage count of the sperm cells showing AR-pattern following exposure to 0.5–20 ppm of lead acetate after 3 h. Similarly, AR-pattern was significantly (p < 0.05) higher in 10 and 20 ppm of lead acetate treated groups after 3 h compared to the corresponding values in these groups after 15 min.
9. *Effect of lead on total cholesterol content*: Cholesterol levels in different lead treated groups (0.5, 5, 10 and 20 ppm) were found to be 34.14, 32.46, 31.35 and 33.49 mg/dl, respectively after 15 min of exposure (Table 3) and these values were almost comparable to that observed in the control group (33.80 mg/dl). Similarly, cholesterol levels in different lead acetate treated groups did not significantly (p > 0.05) differ from control group value after 3 h.
10. *Effect of lead on cyclic-adenosine monophosphate (cAMP) levels*: Perusal of the data summarized in Table 3 revealed that cAMP (pmol/ml) levels increased in the lead acetate treated groups, both after 15 min and 3 h compared to control but that increased was not statistically significant.
11. *Effect of lead on intracellular Calcium (*_*i*_*Ca*^*2+*^*)*: Data summarized in Table 3 and illustrated in Fig. 9 show intracellular calcium release in sperm cells of control and different lead acetate (0.5, 5, 10 and 20 ppm) treatment groups. Compared to the control, there was significant (p < 0.05) reduction in percentage of sperm cells showing intracellular calcium release in lead acetate treated groups after 15 min of exposure. Compared to 46.44% decrease in control group, there was 20.11% decrease in intracellular calcium release in 20 ppm lead acetate treated group. No significant differences in Ca^**2+**^ release between the sperm cells of control group and 0.5 ppm lead acetate treated group were observed after 3 h. However, compared to the control group, significant decrease in percentage of sperm cells showing iCa^**2+**^ was observed in rest of the lead-treated groups. Thus, with increase in lead exposure, decrease in percentage of sperm cells showing iCa^**2+**^ release was observed. Further, compared to iCa^**2+**^ release after 15 min, significantly (p < 0.05) higher percentage of sperm cells were observed in all the lead treated groups (0.5, 5, 10 and 20 ppm) after 3 h. But no such significant increase was observed in control group (15 min V/S 3 h).
12. *Effect of lead acetate (5 and 20 ppm) on ultra-structures of spermatozoa*
13. *Scanning Electron Microscopy (SEM)*: Effect of two different concentrations of lead acetate (5 and 20 ppm) was studied on sperm cells to unravel ultra-structural damages, if any, using scanning electron microscopy (SEM). Scanning electron micrographs of spermatozoa of control group revealed intact surface morphology at different sampling time points i.e., after 15 min and 3 h (Fig. 10). Sperm cells showed normal oval shaped head with intact acrosomes (Fig. 10B and E) and middle piece showed normal midpiece and neck (Fig. 10C and F) with homogenous plasma membrane throughout the sperm cells. Neck and tail of sperms also had regular morphology, both after 15 min and 3 h.
  Fig. 11 illustrates the surface morphology of sperm cells following exposure to 5 ppm lead acetate for 15 min and 3 h. Perusal of Fig. 11A revealed intact surface structure of sperm cell. However, few sperm cells at this time point (15 min) showed irregular plasma membrane of sperm head (Fig. 11B) and middle piece (Fig. 11C). No other obvious changes were observed in sperm tail. Compared to the effect of 5 ppm lead acetate after 15 min, effect of this concentration of lead after 3 h was more pronounced as at this time point, few spermatozoa showed deformities in head and middle piece including swollen acrosome (Fig. 11E) and wavy middle piece (Fig. 11F). Treatment of sperm cells with higher concentration (20 ppm) of lead acetate produced more deleterious effects on plasma membrane of head and tail even after 15 min of exposure (Fig. 12A). At this time point (15 min), plasma membrane integrity of sperm head was disturbed (Fig. 12B) and tail and plasma membrane were swollen (Fig. 12A and C). Compared to the effect of 20 ppm lead acetate on sperm cells after 15 min, morphological defects in spermatozoa were more severe after 3 h of exposure. Deformities were noticed in head, neck, middle piece and tail of spermatozoa at this time point (3 h). Tail of the sperms appeared swollen (Fig. 12D), neck was detached (Fig. 12E) and focal areas of membrane rupture were also observed on sperm head surface (Fig. 12E). Further, middle piece of sperm cell was observed to be deformed and had membrane fractures (Fig. 12F).

**Table 1:** Effect of in-vitro exposure of spermatozoa to different concentration of lead acetate (0.5–20 ppm) on acrosomal integrity, % of MTP Positive spermatozoa, % Caspase 3 and 7 positives and % Spermatozoa with DNA damage at different time intervals

Treatments	% of Acrosome integrity		% of MTP Positive spermatozoa		% Caspase 3 and 7 positives		% Spermatozoa with DNA damage	
	15 min	3 h	15 min	3 h	15 min	3 h	15 min	3 h
Control	81.82 ± 1.59^a^	68.83 ± 6.02^b^	92.13 ± 3.22^b^	82.42 ± 3.38^b^	16.44 ± 1.23^a^	20.68 ± 2.97^a^	3.76 ± 0.10^a^	4.02 ± 0.04^a^
0.5 ppm	73.22 ± 2.76^aB^	66.68 ± 3.02^Ab^	91.26 ± 2.51^abB^	69.26 ± 6.83^abA^	21.56 ± 1.16^a^	23.07 ± 2.05^a^	5.33 ± 0.33^Ab^	8.00 ± 0.60^bB^
5 ppm	72.98 ± 3.64^aB^	63.81 ± 3.47^Ab^	92.16 ± 2.17^abB^	66.30 ± 6.54^abA^	28.84 ± 1.15^b^	30.54 ± 1.39^b^	5.46 ± 0.18^b^	8.07 ± 1.21^b^
10 ppm	70.55 ± 3.92^aB^	58.50 ± 1.55^Aab^	92.01 ± 2.36^abB^	60.14 ± 7.55^abA^	29.73 ± 3.45^b^	33.13 ± 0.25^b^	5.62 ± 0.031^Ab^	9.88 ± 1.23^bB^
20 ppm	66.52 ± 5.39^aB^	47.01 ± 0.29^Aa^	77.29 ± 5.84^aB^	51.13 ± 7.37^aA^	33.76 ± 1.88^b^	35.29 ± 2.67^b^	7.84 ± 0.43^Ac^	15.79 ± 0.61^cB^

Data presented are mean ± SE of the semen samples of six bucks

Different small superscripts in the columns indicate significant (p < 0.05) differences between the different treatment groups

Different capital superscripts in rows indicate significant (p < 0.05) differences between the different time intervals

**Table 2:** Effect of in-vitro exposure of spermatozoa to different concentrations of lead acetate (0.5–20 ppm) on capacitation like changes at different time intervals

Treatments	% sperm cells showing capacitation at different time intervals					
	15 min			3 h		
	F-pattern	B-pattern	AR-pattern	F-pattern	B-pattern	AR-pattern
Control	94.59 ± 1.82	0.20 ± 0.20	5.20 ± 1.91	83.27 ± 4.49^b^	0.2 ± 0.20	16.53 ± 4.55^a^
0.5 ppm lead acetate	90.71 ± 2.40	0.60 ± 0.24	8.68 ± 2.59	80.9 ± 5.53^b^	0.8 ± 0.20	18.30 ± 5.53^ab^
5 ppm lead acetate	87.80 ± 4.55	0.80 ± 0.20	11.39 ± 4.74	77.64 ± 7.34^ab^	1.00 ± 0.31	21.35 ± 7.60^ab^
10 ppm lead acetate	86.28 ± 3.61^B^	0.80 ± 0.20	12.91 ± 3.80^A^	65.46 ± 6.68^Aab^	1.00 ± 0.31	33.53 ± 6.96^Bab^
20 ppm lead acetate	85.10 ± 1.83^B^	1.00 ± 0.00	13.9 ± 1.83^A^	54.66 ± 5.97^Aa^	1.2 ± 0.20	44.14 ± 6.11^Bb^

Data presented are mean ± SE of the semen samples of six bucks

Different small superscripts in the columns indicate significant (p < 0.05) differences between the different treatment groups

Different capital superscripts in rows indicate significant (p < 0.05) differences between the different time intervals

**Table 3:** Effect of in-vitro exposure of spermatozoa to different concentrations of lead acetate (0.5–20 ppm) on Total Cholesterol (mg/dl), cAMP (pmol/ml), Intracellular Ca^2+^ release in spermatozoa (%), and percent spermatozoa showing immune-localization of TPP (Tyrosine phosphorylated proteins) at different time intervals

Treatments	Total Cholesterol (mg/dl)		cAMP (pmol/ml)		Intracellular Ca^2+^ release in spermatozoa (%)		% Spermatozoa showing immunolocalization of TPP	
	15 min	3 h	15 min	3 h	15 min	3 h	15 min	3.0 h
Control	33.80 ± 1.06^a^	32.83 ± 0.77^a^	0.18 ± 0.02^a^	0.38 ± 0.07^a^	46.44 ± 0.01^eB^	42.46 ± 0.01^dA^	80.13 ± 0.62^a^	79.04 ± 0.55^a^
0.5 ppm	34.14 ± 1.00^a^	33.52 ± 0.80^a^	0.31 ± 0.05^a^	0.30 ± 0.12^a^	32.70 ± 0.31^dA^	41.86 ± 0.31^ dB^	78.95 ± 0.46^a^	80.55 ± 0.27^ab^
5 ppm	32.46 ± 1.03^a^	33.38 ± 0.73^a^	0.24 ± 0.04^a^	0.25 ± 0.05^a^	30.11 ± 0.31^cA^	37.94 ± 0.63^cB^	80.85 ± 0.52^aA^	83.18 ± 0.19^bB^
10 ppm	31.35 ± 0.38^a^	30.14 ± 0.64^a^	0.17 ± 0.02^a^	0.17 ± 0.05^a^	25.80 ± 0.31^bA^	34.53 ± 0.33^bB^	80.24 ± 0.52^a^	82.10 ± 0.58^b^
20 ppm	33.49 ± 0.86^a^	32.16 ± 2.05^a^	0.24 ± 0.02^a^	0.25 ± 0.05^a^	20.11 ± 0.310^aA^	24.92 ± 0.31^aB^	80.27 ± 0.23^a^	81.66 ± 1.04^ab^

Data presented are mean ± SE of the semen samples of six bucks

Different small superscripts in columns indicate significant (p < 0.05) differences between the different treatment groups

**Fig. 13:**
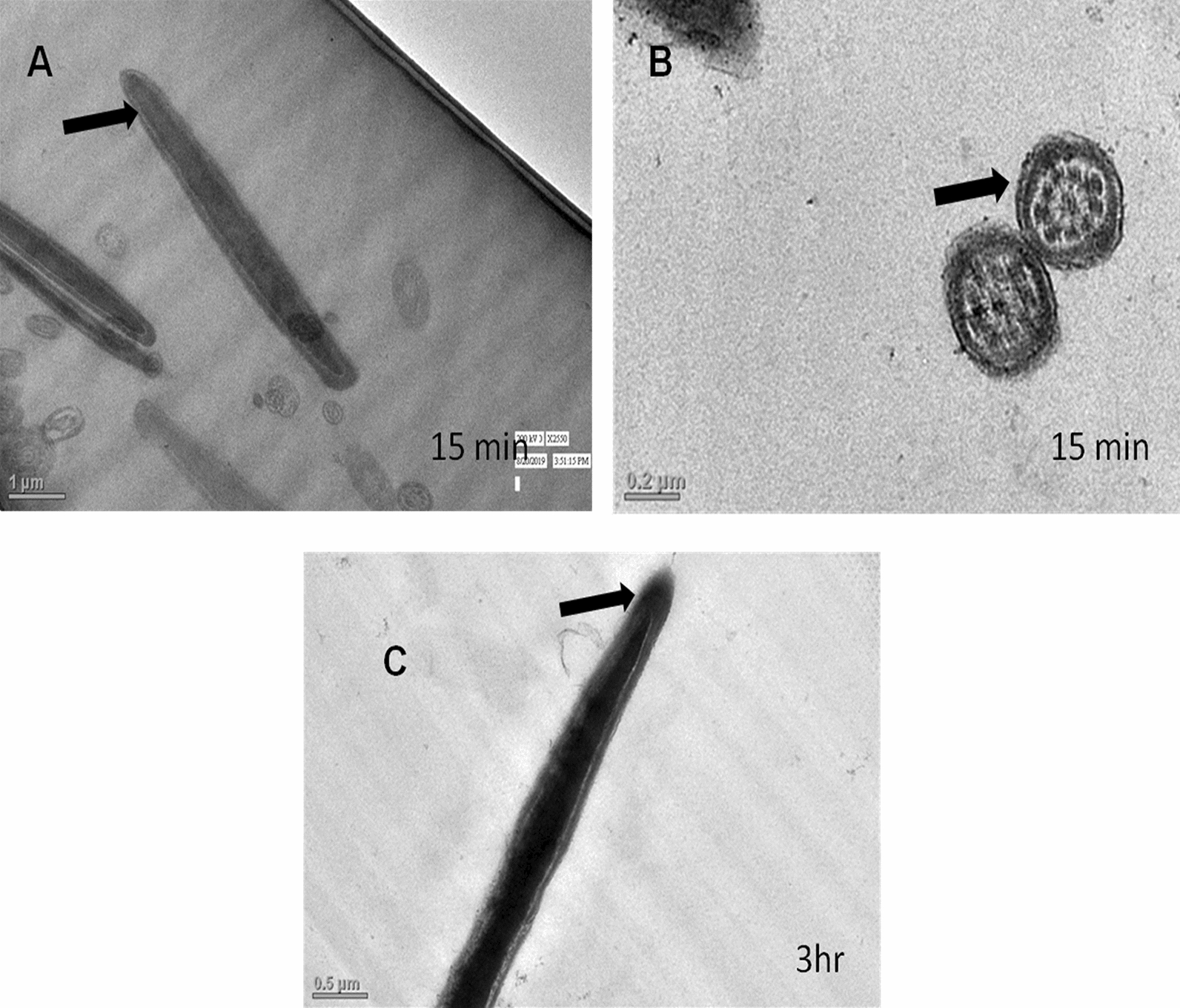
TEM (Transmission Electron Microscopy) images of the buck spermatozoa of control group showing normal ultrastructure i.e., intact plasma membrane (**A**), intact axoneme integrity (**B**) and intact acrosome (**C**)

**Fig. 14:**
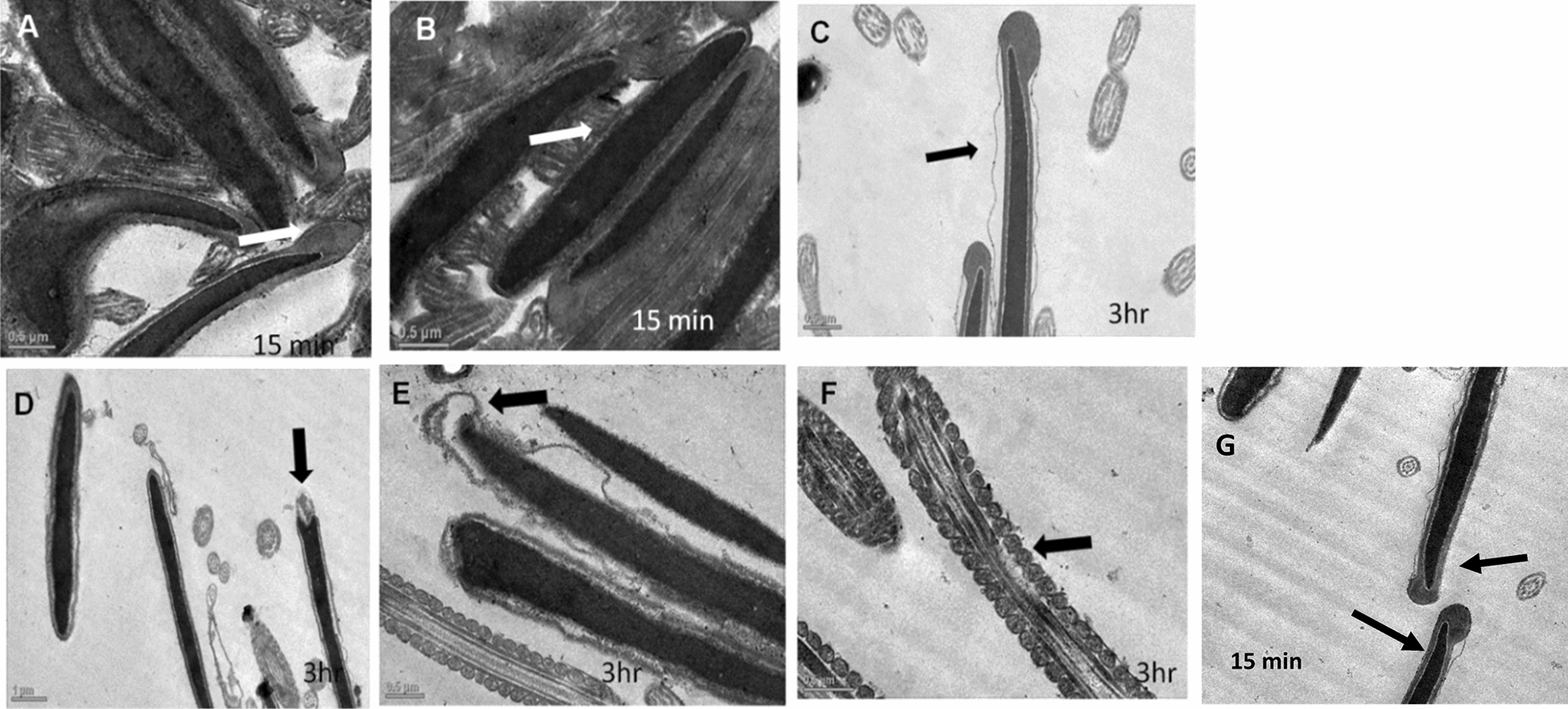
TEM images of buck sperm cells following in vitro exposure to 5 ppm of lead acetate for 15 min and 3 h. Irregular plasma membrane of sperm head (**B**), damaged plasma membrane (**C**) and loss of acrosome content (**D** and **E**) and swollen mitochondria and collapsed cristae in middle piece (**F**). No significant lesions or alterations in tail of the sperms at 15 min time point (**A** and **G**)

**Fig. 15:**
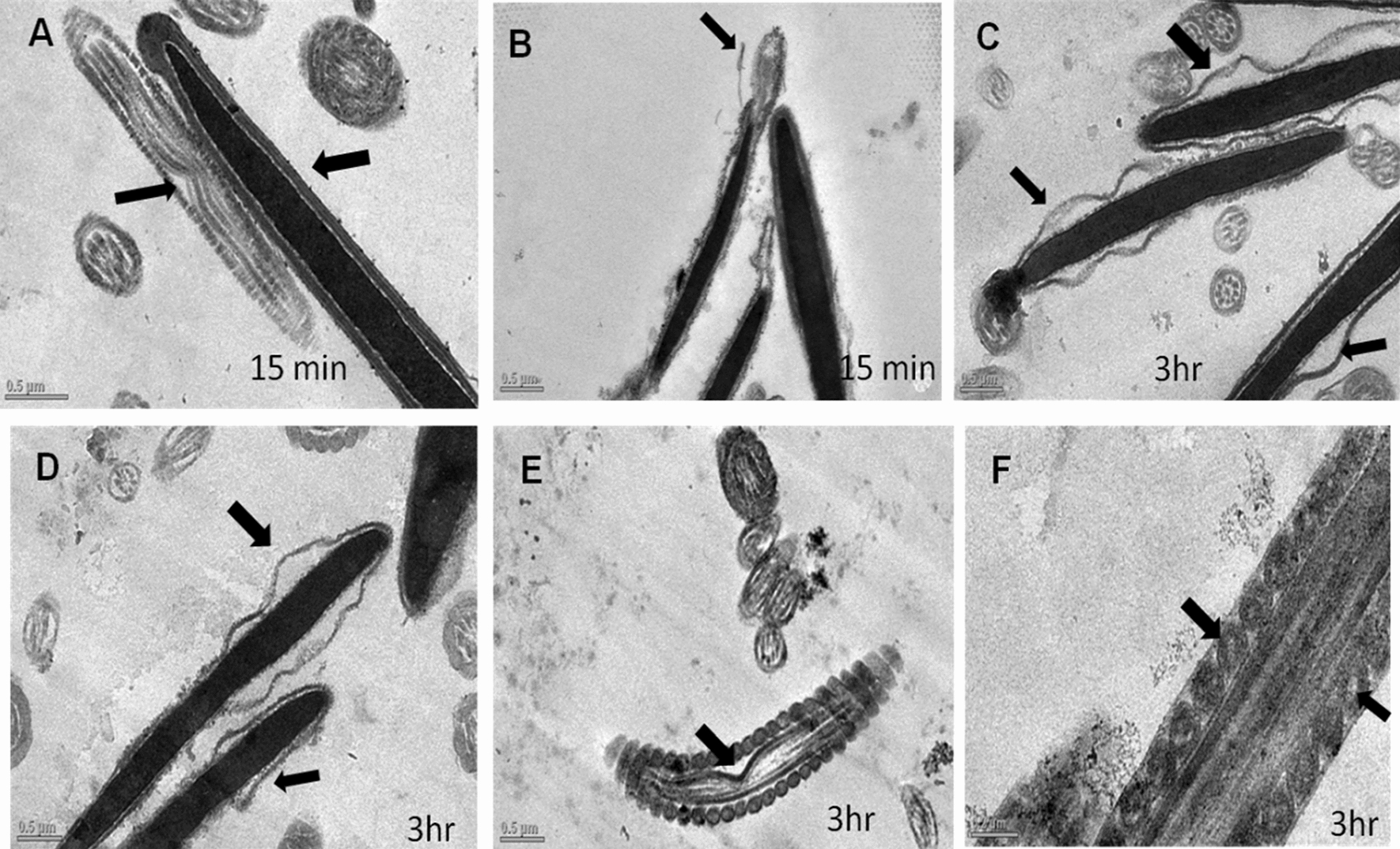
TEM images of the buck spermatozoa following in vitro exposure to lead acetate at 20 ppm level for 15 min and 3 h showing hampered head plasma membrane (**A**), loss of acrosome (**B**), disintegrated plasma membrane (**C**) and focal area lysis (**D**), distorted outer dense fibre (**E**) and collapsed mitochondrial cristae (**F**)

**Fig. 16:**
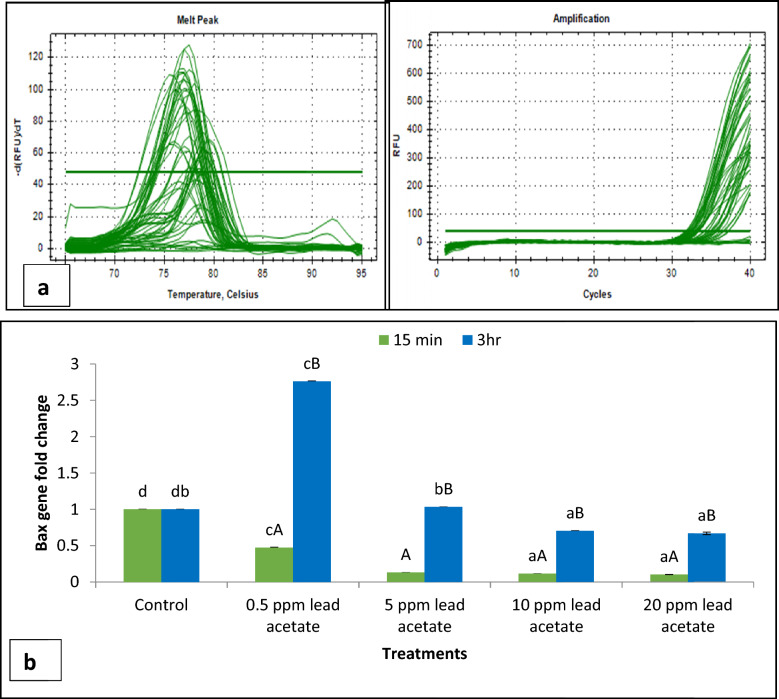
**a** Amplification and melt peak plot of Bax and Beta actin genes in buck spermatozoa treated with different concentrations (0.5, 5, 10 and 20 ppm) of lead acetate after 15 min and 3 h. **b** Fold change in expression of Bax gene in spermatozoa following in-vitro exposure to different concentrations of lead acetate for different time intervals. Data presented are mean ± SE of the semen samples of six bucks. Different capital superscripts on bars indicate significant (p < 0.05) differences between the different time intervals while small superscripts on bars indicate significant (p < 0.05) differences between different concentration of lead

**Fig. 17:**
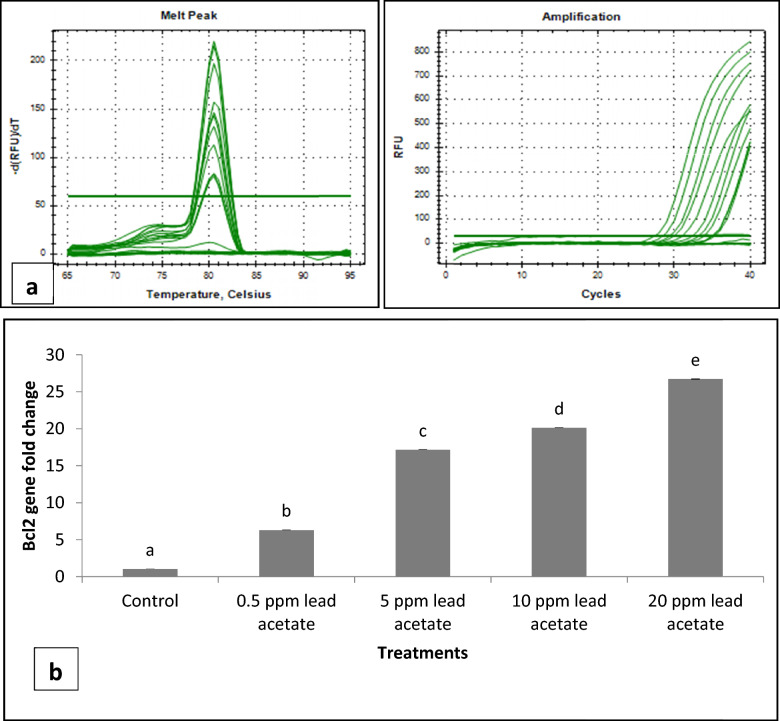
**a** Amplfication and Melt peak plot of Bcl-2 gene in buck spermatozoa treated with different concentrations (0.5, 5, 10 and 20 ppm) of lead acetate after 15 min and 3 h. **b** Fold change in Bcl-2 gene expression in buck spermatozoa following in-vitro exposure to different concentrations of lead acetate for 15 min. Data presented are mean ± SE of the semen samples of six bucks. Different capital superscripts on bars indicate significant (p < 0.05) differences between the different time intervals while small superscripts on bars indicate significant (p < 0.05) differences between different concentration of lead

**Fig. 18:**
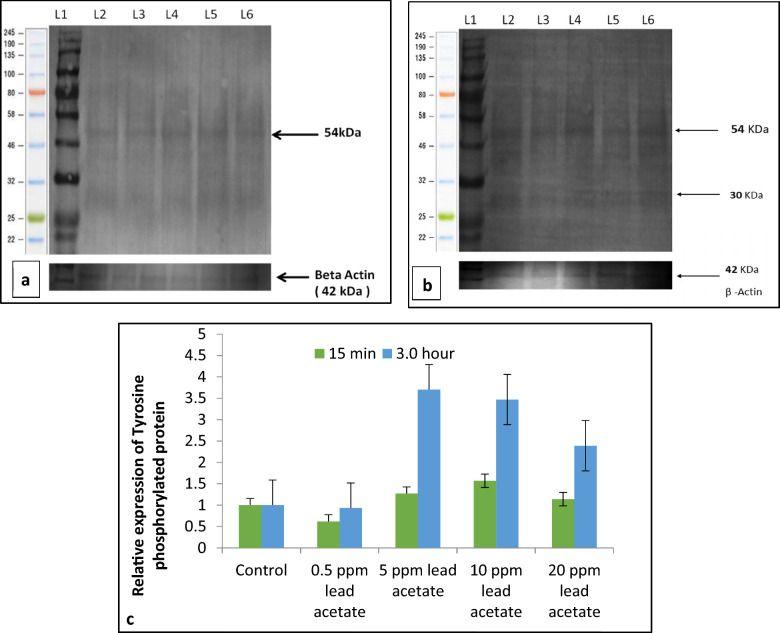
**a** Immunoblot showing the tyrosine phosphorylated protein (54 kDa) in sperm cells of the control and lead acetate (0.5–20 ppm) treated groups after 15 min of exposure. (LI—M.W marker L2—Control L3—0.5 ppm L4—5 ppm L5—10 ppm and L6—20 ppm). **b**: Immunoblot showing tyrosine phosphorylated proteins (54, and 30 KDa) in sperm cells of the control and lead acetate (0.5–20 ppm) treated groups after 3 h of exposure. (L1—M.W marker, L2—Control L3—0.5 ppm L4—5 ppm L5—10 ppm and L6—20 ppm). **c** Quantification of relative expression of tyrosine phosphorylated proteins (western blots) in sperm cells following in vitro exposure to different concentrations of lead acetate (0.5–20 ppm) for 15 min and 3 h

**Fig. 19:**
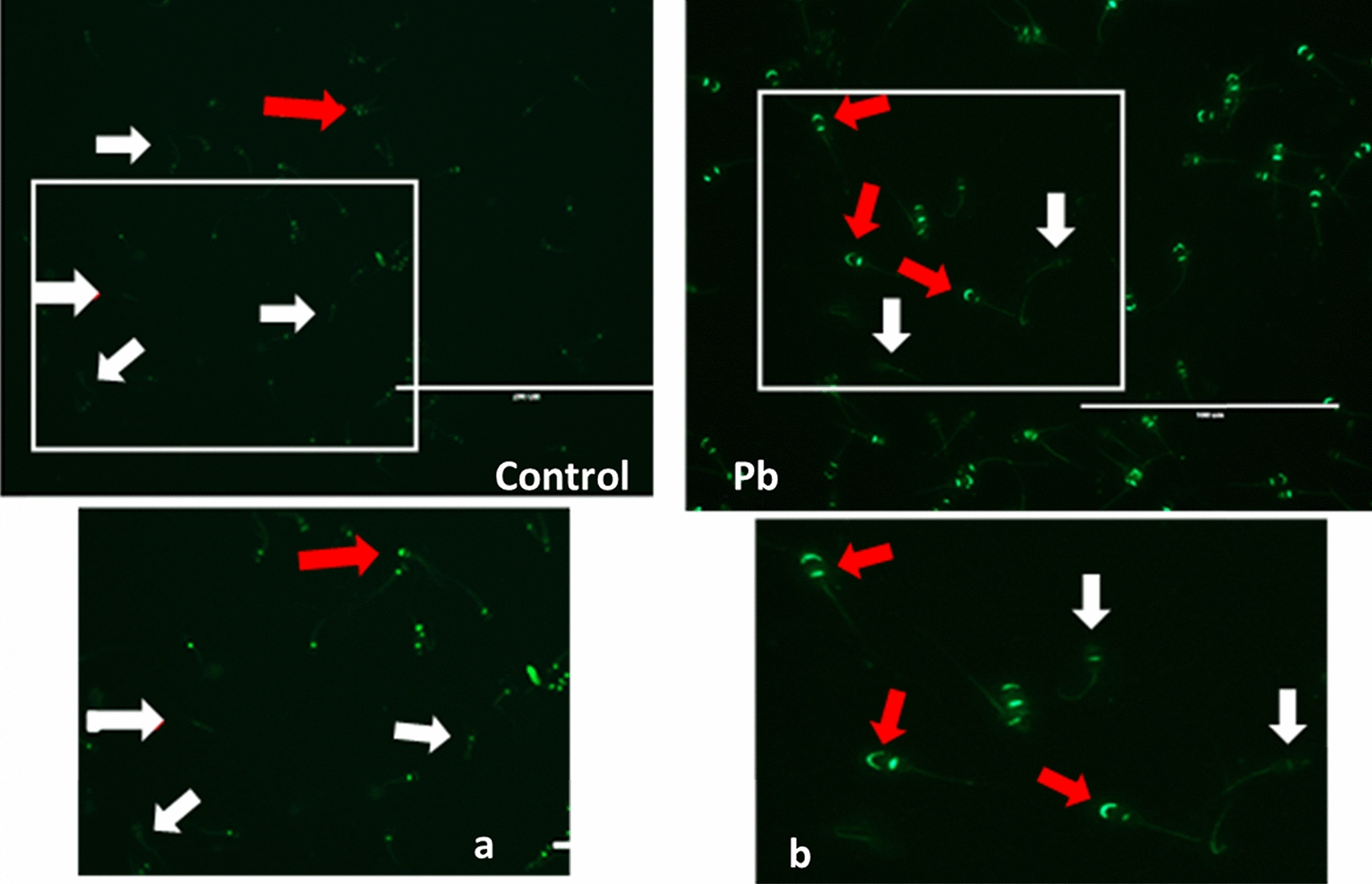
Immuno-localization of tyrosine phosphorylated proteins in sperm cells of control (a; zoom image) and lead acetate (Pb) treated (b; zoom image) groups after 3 h. Localization of tyrosine phosphorylated proteins in pre-acrosomal cap and post-acrosomal region are indicated by red arrow while no/less immuno-localization of tyrosine phosphorylation indicated by white arrow

Different capital superscripts in rows indicate significant (p < 0.05) differences between the different time intervals

ii. *Transmission Electron Microscopy (TEM)*: Ultra-structural analysis of TEM images of buck spermatozoa revealed intact plasma membrane of sperm cells of the control group (Fig. 13A). Axoneme integrity in this group, appeared intact (Fig. 13B) and majority of spermatozoa of this group showed intact acrosome (Fig. 13C), intact head and tail plasma membrane (Fig. 13A). Just like SEM observations in 5 ppm lead acetate treated group after 15 min, ultra-structural analysis of the TEM images also revealed irregular plasma membrane of spermatozoa head (Fig. 14A). But there were no marked lesions or alterations in tail of sperms at this time point (Fig. 14G). Exposure of sperm cells to 5 ppm lead acetate for 3 h resulted in marked deleterious effects as longitudinal sections of spermatozoa showed rupture of plasma membrane and loss of acrosome content from sperm head (Fig. 14C–E). Mitochondrial deformities and swollen mitochondria having collapsed cristae in middle piece were observed in some of spermatozoa (Fig. 14F). Longitudinal sections of spermatozoa after exposure to 20 ppm lead acetate revealed that head plasma membrane and tail of spermatozoa were affected even after 15 min of exposure (Fig. 15A and B). However, many spermatozoa at this time point had intact ultrastructures. Some of the spermatozoa showed distorted outer dense fibre in middle piece (Fig. 15A). Sperm head plasma membrane and acrosome integrity were affected after 15 min exposure to 20 ppm lead acetate (Fig. 15B). Spermatozoa with reacted acrosome were also noticed at this time point in 20 ppm lead-treated group (Fig. 15B). Compared to the ultra-structural changes in 20 ppm lead-treated group after 15 min, these were more pronounced after 3 h and were in accordance with the SEM observations as deformities in head, middle piece and tail of sperm cells were noticed at this time point as well. Longitudinal sections of the head revealed disintegrated plasma membrane and focal areas of lysis (Fig. 15C and D). Deformities in middle piece region included distorted outer dense fiber (Fig. 15E) and collapsed mitochondrial cristae (Fig. 15F).
iii. *Comparative expressions of apoptotic (Bax) and anti-apoptotic (Bcl-2) genes*: Compared to the control group, relative mRNA expression data of Bax gene (apoptotic) in sperm cells was significantly (p < 0.05) decreased in lead acetate treated (0.5, 5, 10 and 20 ppm) groups after 15 min of exposure and this effect was concentration-dependent. Similar significant (p < 0.05) alterations in relative mRNA fold change expression of Bax gene were observed in semen samples pretreated with lead acetate (0.5, 5, 10 and 20 ppm) for 3 h (Fig. 16b) were also observed. Expression of Bax gene was found to be highest (2.76 ± 0.01) and significantly increased in 0.5 ppm of lead acetate group while significantly decreased in 10 and 20 ppm of lead acetate treated groups after 3 h. Further, compared to 15 min, fold change expression of Bax gene was significantly (p < 0.05) increased within all lead treated groups at 3 h i.e., as the time of exposure to lead increased, there was increased expression of the Bax gene. Compared to control, fold change expression of of Bcl-2 gene in semen samples increased significantly (p < 0.05) even after 15 min of exposure to lead acetate (0.5, 5, 10 and 20 ppm). But compared to control, there was no expression of Bcl-2 gene at all in semen samples exposed to different concentrations (0.5, 5, 10 and 20 ppm) of lead acetate for 3 h (Fig. 17b).
iv. *Effect of lead on relative expression of tyrosine phosphorylated proteins*: Capacitation like changes in spermatozoa following exposure to lead were evaluated by determining the phosphorylation of tyrosine containing proteins in sperm lysates of control and lead acetate treated groups using immune blotting and immune fluorescence methods and results of the same have been shown in Fig. 18a and b. Immuno-blot confirmed the presence of 54 kDa at 15 min and while 30 and 54 kDa tyrosine phosphorylated proteins after 3 h of lead exposure. Relative quantification of the expressions of tyrosine phosphorylated proteins in different lead acetate treated groups (0.5, 5, 10 and 20 ppm) were found to be 0.62, 1.27, 1.57 and 1.14 respectively, after 15 min compared to control. Compared to control, the relative expression was higher in 5, 10 and 20 ppm lead acetate-treated groups while lesser in 0.5 ppm lead acetate treated groups after 15 min of exposure. Further, the relative expression (Fig. 18c) in 20 ppm lead acetate group was lower than in 5 and 10 ppm lead acetate-treated groups. After 3 h, compared to control, relative expressions of tyrosine phosphorylated proteins in different lead acetate (0.5, 5, 10 and 20 ppm) treated groups were found to be 0.93, 3.70, 3.47 and 2.39, respectively. Critical perusal of the data revealed that relative expression was higher in 5, 10 and 20 ppm lead acetate-treated groups while lower in 0.5 ppm treated group. Further, compared to 5 and 10 ppm lead treated groups, it was comparatively lower in 20 ppm lead–treated group.
v. *Effect of lead on immunolocalization of tyrosine phosphorylated proteins (TP)*: Indirect immune-fluorescence assay was used for immuno-localization of TP in semen samples. Positive immuno-reactivity was observed in pre-acrosomal cap and post acrosomal region of spermatozoa (Fig. 19) indicating localization of TP proteins pre-dominantly in these two regions of sperm cells in control and different lead treatment groups. Apparently, percentage of spermatozoa showing immunolocalization of TP in pre-acrosomal region was higher than in the post acrosomal region. But flagella of the sperm cells did not show any positive immune reactivity.

Spermatozoa showing immunolocalization of tyrosine phosphorylated proteins in control group did not significantly differ from those observed in lead (0.5–20 ppm) treated groups after 15 min of exposure. However, immunolocalization was found to be significantly (p < 0.05) higher in 5 and 10 ppm lead acetate treated groups after 3 h of exposure. Further, percentage of spermatozoa showing immunolocalization of TP proteins was significantly (p < 0.05) higher after 3 h (83.18 ± 0.19 %) compared to that observed after 15 min (80.85 ± 0.52 %) in 5 ppm lead treated group. But in rest of the lead-treated groups including control group, no significant differences were observed between the values after 15 min and 3 h.

**Fig. 20:**
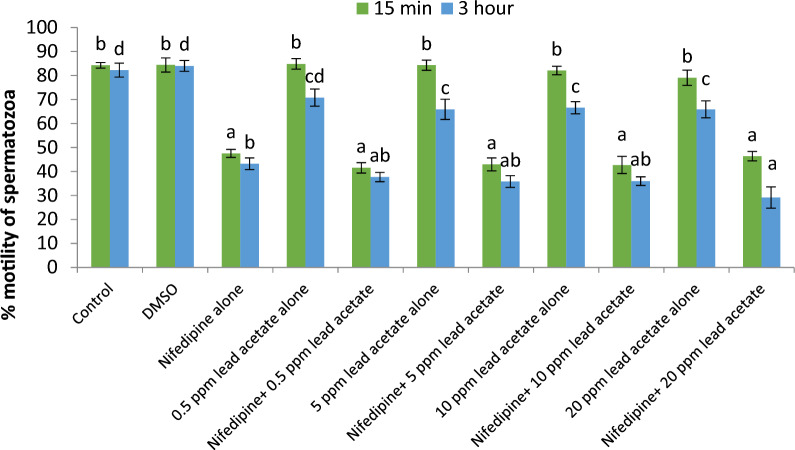
Motility (%) of spermatozoa following *in-vitro* exposure to different concentrations of lead acetate alone and lead acetate in the presence of nifedipine (500 μM) at different time intervals. Data presented are mean ± SE of the semen samples of six bucks. Vertical bars represent the standard error (SE). Different small superscripts on bars indicate significant (p < 0.05) differences between the different treatment groups at different time intervals

**Fig. 21:**
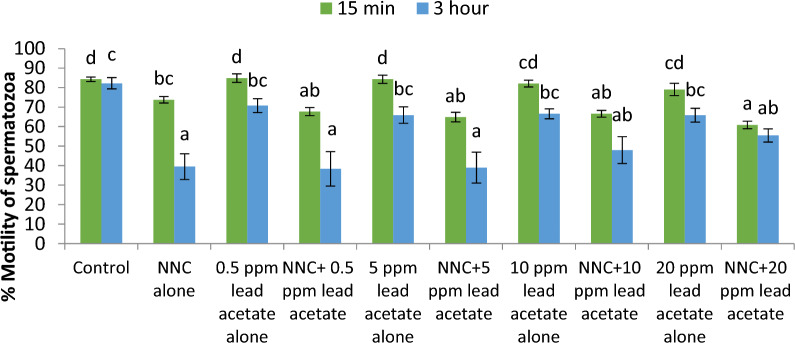
Motility (%) of spermatozoa following *in-vitro* exposure to different concentrations of lead acetate alone and in the presence of NNC (10 μM) at different time intervals. Data presented are mean ± SE of the semen samples of six bucks. Vertical bars represent the standard error (SE). Different small superscripts on bars indicate significant (p < 0.05) differences between the different treatment groups at different time intervals

16. *Effect of lead acetate on Ca*^*2+*^* channels*
17. *Effect of Nifedipine (L-type Ca*^*2+*^* channels blocker) on sperm motility*: Data summarized in Fig. 20 shows the effect of different concentrations of lead acetate (0.5, 5, 10 and 20 ppm) alone and lead acetate in the presence of nifedipine (500 μM), a L-type Ca^2+^ channel blocker, after 15 min of pre-exposure. At the 15-min time interval, the control group exhibited a total motility of spermatozoa of 84.27 ± 1.15%. Treatment with 0.5% DMSO did not significantly affect motility (84.39 ± 2.92%) compared to the control. However, nifedipine alone significantly reduced the sperm motility to 47.51 ± 1.65% showing a decrease of 43.62%. Lead acetate exposure alone at concentrations of 0.5, 5, 10, and 20 ppm did not significantly impact motility compared to the control group. Notably, the combination of nifedipine and lead acetate at all tested concentrations (0.5, 5, 10, and 20 ppm) resulted in a significant (p < 0.05) decrease in motility, ranging from 41.52 ± 2.18% to 46.37 ± 1.96%, corresponding to reductions of 50.73% and 44.97%, respectively. Total motility of sperm cells in control (82.25 ± 2.90%), and DMSO treatment (84.01 ± 2.26%) groups were not altered even after 3 h. Compared to the control groups, moderate reduction in motility, ranging from 65.90 ± 3.53% to 70.78 ± 3.57% showing 13.95–19.88% decrease, was observed in different lead acetate exposure alone groups (0.5–20 ppm). But significant reduction (47.49%) in spermatozoa motility was observed only in nifedipine alone treated group. In nifedipine pretreated sperm cells, further decrease in percent motility of sperms was not observed when exposed to different concentrations of lead (0.5, 5, 10, and 20 ppm). Thus, suggesting that lead was not able to further reduce the motility of sperm cells once the L-type Ca^2+^ channels were blocked. Thus, lead apparently seems to either interfere with Ca^2+^ influx mechanism or mimics Ca^2+^ and enters through L-type Ca^2+^ channels but does not perform Ca^2+^ functions in sperm cells.
18. *Effect of NNC 55-0396 hydrate (T-type Ca*^*2+*^* channels blocker) on sperm motility*: Data illustrated in Fig. 21 shows the motility of spermatozoa at different time intervals following exposure to different concentrations of lead acetate (0.5, 5, 10 and 20 ppm) alone and in the presence of NNC (10 μM), a T-type Ca^2+^ channel blocker. Spermatozoa of control group exhibited a total motility of 84.27 ± 1.15% after 15 min. Compared to control group, no significant decrease in motility of sperms was observed in lead acetate alone (0.5–20 ppm) treated groups. But significant (p < 0.05) decrease in motility (73.79 ± 1.68%) was observed in NNC (10 μM) alone treatment group at this time point. It was interesting to note that in NNC-pretreated semen samples, lead was found to augment reduction in percent motility (60.88 ± 1.93% − 67.68 ± 2.07%) and this inhibitory effect was statistically significant. After 3 h, sperms of NNC alone treated group exhibited 39.47 ± 6.58% motility vis-a-vis 82.25 ± 2.90% in control group and reduction was 52.01% and statistically significant. Lead exposure alone (0.5–20 ppm) for 3 h resulted in moderate reduction in motility which ranged between 65.90 ± 3.53% and 70.78 ± 3.57%. Notably in NNC pre-treated sperms, lead acetate at different concentrations (0.5–20 ppm) resulted in a further decrease in total motility, ranging from 38.33 ± 8.85% to 55.47 ± 3.40% compared to the effect of NNC alone after 3 h. Thus, revealing that on blockade of T-type channels, lead produced more pronounced inhibitory effect on sperm motility both after 15 min and 3 h and suggesting the presence and involvement of T-type Ca^++^ channels in regulating sperms motility and Pb^2+^ interacts or affects some other cellular or molecular sites and inhibits sperms motility.

## Discussion

Present study was undertaken to unravel the mechanistic signaling pathway(s) of toxic effects of lead on kinematics and functional dynamics of goat spermatozoa based on molecular and ultrastructural evidence(s). Sperm motility is one of the most critical indicators of semen quality and determinant of male fertility and fertilization success [25]. Our results evidently suggest lead-induced decrease in progressive sperm motility, alterations in kinematic patterns and concentration and time-dependent killing effects of lead on spermatozoa are in agreement with the reported correlation between concentration of lead in blood and impaired total and progressive motility of sperms in humans [26–29]) and wild bird *Alectoris rufa* [30]. Similarly, an association between increase in testicular lead levels and damage to sperm chromatin and sperm membrane integrity in red deer [31] and significant decrease in sperm viability, membrane integrity and motility has been reported in rats [32]. But our observations on buck-spermatozoa or those reported in rats or red deer are contrary to effect of lead acetate on in vitro exposure (10–100 µM) in human sperms where lead failed in significantly altering viability of sperm cells [26]. Although difficult to put forth any plausible explanation for this baffling observation but the possibility of shorter exposure period cannot be ruled out.

Hypo-osmotic swelling (HOS) test is considered as gold standard to assess the viability of sperms in fresh semen [33]. Membrane integrity of goat spermatozoa was significantly (p < 0.05) reduced in 20 ppm lead acetate treated group while moderately to markedly in 0.5–10 ppm lead acetate treated groups after 3 h. Our observations are in the present study are in agreement with significant reduction in sperm viability and HOST percentage in battery and paint factory workers [34]. Fertility in male depends on structurally and biochemically intact acrosome in spermatozoa head. During acrosome reaction, hyaluronidase is released which digests cumulus oophorus cells and acrosine helps in dissolution of zona pellucida [35] and allows penetration of the sperm head and results in sperm-oocyte fusion. Spermatozoa with damaged acrosome do not show any acrosome reaction and thus there are fertilization failures ([36, 37]. Our findings in the present study suggest that acrosomal integrity of sperm cells was adversely affected by lead in concentration- and time–dependent manner. Vulnerability of goat sperm cells to lead-induced acrosome damage in the present study is in agreement with positive correlation between lead levels and spontaneous acrosome reaction in *in-vitro* experiments in humans [38] and mice [39]. Likewise, cadmium has also been reported to induce spontaneous acrosome reaction in spermatozoa of mouse [39, 40], ram [41], rabbit [42] and sheep [43]. Based on similar findings, Oliveira et al. [39] had suggested lead-induced premature acrosome reaction in sperms and compromised physiological functions (motility and fertilizing ability) of sperm cells.

Lead–induced compromised cellular mitochondrial functions like inhibition of mitochondrial oxidative phosphorylation, decrease in mitochondrial membrane potential MTP), inhibition of mitochondrial respiratory enzyme activities, ATP depletion, and energy crisis have been reported by several researchers [44–48]. In the present study, MTP of spermatozoa was significantly reduced even after 15 min of lead exposure and percentage of high MTP positive spermatozoa significantly decreased with increase in exposure level from 0.5 to 20 ppm and exposure time from 15 min to 3 h. Thus, lead-induced impairment of sperm mitochondrial function may be responsible for decrease in progressive motility and hyperactivation of sperm cells. Our observation is in confirmation with the recent report about lead-induced impairment in sperm mitochondrial function through increase in mitochondrial depolarization and decline in mitochondrial dehydrogenase activity [32]. Similar effect of mercury on MTP in buck spermatozoa has recently been reported from our laboratory as well [24].

Apoptosis is accompanied by sequence of characteristic biochemical changes, including mitochondrial outer membrane permeabilization (MOMP), activation of effector caspases (caspase 3, caspase 6 and caspase 7), and activation of catabolic hydrolases which degrade most of the macromolecules of cells, including DNA [49]. Caspase activation is the final process of death signalling pathway, in which procaspase-3 is activated to caspase-3, which is the most important biomarker and executor of cell apoptosis. However, cells can survive direct or transient caspase-3 activation. High doses of caspase uniformly result in cell death and low doses in cell survival while intermediate caspase activity has been reported to be compatible with either of the outcomes [50]. Thus, in our study 5 ppm lead on 15 min exposure possibly resulted in transient caspase-3 activation to enhance cell repair after lead-induced injury but lead at higher concentration (20 ppm) on prolonged exposure promoted apoptosis which was not reversible. Our observations on alteration in caspase expression in buck spermatozoa are in confirmation with concentration-dependent significant increase in caspase-3-like activity in PC 12 cells following exposure to lead acetate (0.1, 1 and 10 µM) for 24 h [46].

The Bcl-2 gene family members, including Bax and Bcl-2, play essential role in regulating apoptosis by forming hetero- and homodimers in mitochondrial membrane, and the outcome depends on ratio of Bax to Bcl-2 [51]. The Bax/Bcl-2 ratio acts as rheostat to determine susceptibility of cells to apoptosis [52]. Concentration-dependent significant decrease in expression of pro-apoptotic Bax gene in sperm cells was observed in the present study following exposure to lead for 15 min. Contrary to the effect after 15 min exposure, expression of Bax gene was significantly increased in 0.5 ppm lead treated group while significantly decreased in 10 and 20 ppm lead-treated groups after 3 h of exposure. Further, contrary to the decrease in expression of Bax gene after 15 min of exposure, expression of the anti-apoptotic Bcl-2 gene was significantly increased,and interestingly absolutely no expression of Bcl-2 gene was observed in any of the lead-treated-groups after 3 h. Significant and concentration-dependent decreases in ratio of the expression of Bax/Bcl-2 genes were observed even after 15 min of exposure to lead acetate (0.5–20 ppm). Therefore, our Bax/Bcl-2 ratio and caspase-7 and 3 expressions data suggest that initially sperms strive to resist lead (0.5–20 ppm)-induced apoptosis but on prolonged exposure (3 h) lead promotes apoptosis which may be responsible for decreasing viability of sperm cells. Our findings are in line with lead-induced DNA damage, up-regulation of Bax and down-regulation of Bcl-2 genes and apoptosis in PC 12 cells [46] and nickel–induced decrease in Bcl-2 protein expression and increase in Bax protein expression through suppression of PI3K/Akt pathway in liver of Kunming mice [53].

Sperm DNA damage, which includes DNA denaturation and DNA fragmentation (SDF), is the major cause of defective sperm function and infertility, recurrent spontaneous abortions, pre- and post-implantation losses, accelerated aging, and childhood cancer [54]. Lead produced concentration- as well as time-dependent significant increase in percentage of TUNEL positive spermatozoa indicating damage in DNA of buck sperms. Even the lowest used level of lead (0.5 ppm) within 15 min of exposure resulted in significant damage to DNA which significantly increased with increase in exposure time from 15 min to 3 h. Our observations on lead-induced DNA damage in buck spermatozoa are in agreement with significant increase in DNA fragmentation in human spermatozoa after in vitro exposure to 30 μg/ml of lead chloride for 4 h [55], increased chromatin fragmentation and reduced acrosome integrity in spermatozoa of red deer from lead polluted areas [31] and significant (p < 0.05) increase in percentage of fluorescein isothiocyanate (FITC)-positive sperm cells (DNA damaged) in mice treated with 0.25%, 0.5%, and 1.0% lead acetate [56].

cAMP and Ca^2+^ are the most important second messengers which play an important role as signalling molecules in modulating mammalian sperm functions by modulating capacitation [57]. cAMP levels were found to be increased in all lead acetate treated groups after 15 min of exposure, but the increase was not statistically significant. Contrary to increase in cAMP levels after 15 min, cAMP levels were found to be decreased after 3 h. Similar lead-induced dose-dependent decrease in sperm intracellular cAMP level has also been reported in humans [26]. Increase in adenylate cyclase activity in cerebellum and brain stem after 1 h while significant decrease after 4 h of lead acetate injection (25 mg/kg, i.v.) has been reported in rats by Ewers and Erbe [58]. Lead-induced decrease in intracellular cAMP has been attributed to inhibition of adenylate cyclase activity [59, 60]. Therefore, initial increase followed by decrease in cAMP levels in lead-treated sperm cells may possibly be due to differential modulation of adenyl cyclase activity following exposure for 15 min or 3 h. But to substantiate this, further studies are warranted on effect of different concentrations of lead after exposure for different time intervals.

Ca^2+^ plays important role in modulation of mammalian sperm function [61]. Changes in levels of cAMP and Ca^2+^ trigger series of intracellular signals, such as tyrosine phosphorylation, and regulate sperm functions [62]. Lead acetate significantly reduced percentage of sperm cells showing intracellular calcium release in all the lead acetate-treated groups (0.5–20 ppm) both after 15 min and 3 h of exposure and this effect was concentration-dependent. We used calcium-free media in the present study, but still observed lead-induced premature or spontaneous acrosomes reactions in sperm cells. Recently, similar effect of mercury has been reported from our laboratory on in vitro exposure of buck spermatozoa [24]. Our observation on lead-induced decrease in intracellular Ca^2+^ release in buck sperm cells was similar to the findings of He et al. [26], where sperm [Ca^2+^]i level was reduced by lead acetate (2.5–100 μM) within one min of exposure, and this inhibition was intensified with increase in lead concentrations up to 50 μM at which around 50% inhibition was observed. However, He et al. [26] had used synthetic human tubal fluid (HTF) medium (with 3 mM calcium outside the cell). But in our study, we had used Ca^2+^ free phosphate buffer solution as the medium. Lead is reported to inhibit voltage-gated calcium channels in neuron cells and competes with calcium uptake [63]. Therefore, possibility of reduced intracellular calcium levels in lead-exposed sperms due to reduced permeability of sperm plasma membrane channels to Ca^2+^ in the presence of lead or reduced mobilization of calcium from internal organelles (mitochondria/acrosome) cannot be ruled out. Altered intracellular Ca^2+^ and cAMP levels, and increased pH result in tyrosine phosphorylation of sperm membrane proteins during capacitation [64–66] and sperms undergo a change in motility pattern, which is termed as hyperactivation called beat cross frequency (BCF) [67]. Lead-induced decrease in intracellular Ca^2+^ may be responsible for compromised capacitation, beat cross frequency (BCF) and tyrosine phosphorylation in sperms.

Chlortetracycline (CTC) fluorescence assay data showing effect of lead on capacitation of sperms revealed significant decrease in percentage spermatozoa showing F pattern after exposure to 20 ppm lead acetate for 3 h. Almost concentration-dependent marked to significant increase in number of sperm cells showing AR pattern was also observed after exposure to lead for 3 h. Therefore, our findings evidently suggest that lead either induces very rapid capacitation or the sperm cells straight away undergo acrosome reaction. But to precisely unravel whether there is rapid capacitation or direct acrosomal reaction, further studies are warranted by taking more frequent observations, may be at 3–5 min time intervals. Our findings are in confirmation with lead-induced early onset of capacitation through ROS generation in rat spermatozoa, and resultant premature acrosome reaction and reduced zona-intact oocyte-penetrating capability of sperms [68]. Benoff et al. [38] have also reported positive correlation between lead levels and spontaneous acrosome reaction based on in-vitro studies. Under physiological conditions, influx of extracellular Ca^2+^ through plasma membrane into the outer acrosomal membrane inactivates membrane ATPases during capacitation ([69–71], and initiates acrosomal reaction (AR) by series of point fusions between plasma membrane and the underlying outer acrosomal membrane [72]. Permeability of Ca^2+^ channels to lead is much higher than Ca^2+^, therefore, lead (Pb^2+^) could compete with Ca^2+^ for uptake mechanism via Ca^2+^ channels. But after passage of Pb^2+^ through Ca^2+^ channels, these channels do not become inactive [73], thus, results in persistent inactivation of ATPases which ultimately result in AR [72]. Lead also inhibits the activity of ATPases by binding to SH-group [73, 74].

Motility of sperm cells largely depends upon intactness of ion channels and their involvement in regulating ionic gradient, which in turn regulate motion characters of spermatozoa. Inhibitory effect of lead on spermatozoa motility can be attributed to its ability to rapidly pass through the voltage operated calcium channels (VOCC) and then allowing the VOCCS to remain open or result in hyperpolarization state [73, 75], and thus decrease in sperm motility. Lead-induced decrease in motility in human sperms following exposure to lead has been reported by Hosni et al. [76]. Nifedipine, a known selective L-type calcium channel blocker, is known to significantly induce non-competitive inhibition in uptake Ca^2+^and decrease the activity of Ca^2+^-dependent ATPase enzyme [77]. Apart from importance of Ca^2+^ for enzymatic activity, motility of spermatozoa is also dependent on availability of Ca^2+^ as following exposure of sperm cells with nifedipine, pattern of motility changed within two hours from rapid and linear progression to slow or sluggish linear or non-linear movement and finally to non-progressive motility or even immobility [77]. Thus, implying the significance of Ca^2+^ and Ca^2+^ channels in modulating motility of spermatozoa. We in our study observed that on closure of L-type of Ca^2+^ channels, motility of buck-spermatozoa was significantly reduced and in nifedipine pre-treated semen samples, lead did not further reduce the total motility of sperm cells after 15 min. But lead acetate at 20 ppm in the presence of nifedipine after 3 h significantly reduced total motility of sperm cells. It could be due to direct toxicity of lead on sperm cells. Thus, implying that lead either acts by inhibiting the availability of sufficient intracellular calcium required for motility of sperm cell or decreased the activity of Ca^2+^ dependent ATPase; and resulted in disturbed Ca^2+^ homeostasis along with altered local intracellular Ca^2+^ dynamics and adverse effect on downstream signaling pathway [78] in spermatozoa. Nguyen et al. [79] observed significant reduction in motile sperms count following 5 min incubation of spermatozoa with different Ca^2+^ channel inhibitors. But there were no significant differences on sperm motility between the effects of L–type channel inhibitor (nifedipine), R–type channel inhibitor (SNX–482) and SOC inhibitor (MRS–1845). Therefore, our results on effect of lead and/or nifedipine are in agreement with the observations of Nguyen et al. [79] that nifedipine significantly reduced the motility of sperm cells. But they had not studied any interaction between calcium channel blockers and any metal. Therefore, not possible to compare our findings with any report in the literature.

Lishko et al. [80] have shown that NNC (2 μM) inhibited the progesterone-activated calcium current through human CatSper. Similar to the observations of Nguyen et al. [79] that nifedipine significantly reduced motile sperms count, Tamburrino et al. [81] also observed NNC (10 μM) induced decrease in total motility of sperm by 5–10%. In the present study we also observed significant decrease in total motility of spermatozoa following exposure to NNC for 15 min and 3 h. Our findings on effect of NNC on buck sperm cells are in agreement with the observations of Tamburrino et al. [81]. All these findings, including our, evidently suggest presence and functional involvement of T-type calcium channel in modulating motility and velocity of buck spermatozoa motility. Lead potentiated the inhibitory effect of NNC alone on motility of sperm cells. Interestingly and contrary to effect of lead in the presence of nifedipine, lead markedly reversed the inhibitory effect of NNC on total motility at 10 and 20 ppm lead levels. Thus implying that lead at higher concentrations reversed the inhibitory effect of NNC probably by mimicking Ca^2+^ and entering into the cell possibly via L-type channels or/and facilitating release of Ca^2+^ from intracellular storage sites as a compensatory mechanism to maintain the vitality and motility of the spermatozoa as has been suggested by Kirichok et al. [82] and Alasmarie et al. [83] that in addition to Ca^2+^ current through CatSper channel, Ca_i_^2+^ might be provided from some other sources. It is also possible that apart from Ca^2+^, certain other factors such as cAMP, intracellular alkalization and tyrosine phosphorylation might control motility [84].

To fertilize oocyte, sperm membrane must undergo cholesterol efflux during capacitation, either in the female reproductive tract or in vitro [85]. Capacitation onset rate in sperms principally depends on amount of cholesterol contained in its plasma membrane [86] and cholesterol efflux which results in change in membrane architecture, and increases bilayer permeability and membrane instability, thus, capacitation of sperm cells [87]. In our present study, lead did not significantly influence efflux of cholesterol from membranes of sperm cells up to 20 ppm level. Therefore, possibility of cholesterol efflux-independent acrosomal reaction by lead in buck spermatozoa cannot be ruled out as reported by Nolan et al. [88]. However, further studies are warranted on these aspects.

Phosphorylation of mammalian sperm tyrosine is a critical event in sperm function including its capacitation [89]. Levels of cAMP and Ca^2+^ in sperm cells have been correlated with tyrosine phosphorylation in spermatozoa [62]. The relative expression of tyrosine phosphorylated proteins was lower in 20 ppm lead acetate treated group after 15 min compared to 5 and 10 ppm treated groups. But the relative expression of tyrosine phosphorylated proteins increased in all lead acetate (0.5–20 ppm) treated groups after 3 h. Further, immunolocalization of tyrosine phosphorylation (TP) proteins, a gold standard test, was studied to further validate our findings. Percentage of the spermatozoa showing immune-localization of TP in pre-acrosomal region was higher than in the post acrosomal region in lead-treated (in 5 and 10 ppm) groups compared to control. Thus, our findings suggest an increase in expression of tyrosine phosphorylated proteins with increase in exposure time, but the effect was more pronounced in higher dose groups. Inhibitors of PKA have been reported to block sperm capacitation which results in increase in protein tyrosine phosphorylation [90]. Based on the scientific evidence available in literature and our results, we are tempted to infer that increased tyrosine phosphorylation in sperm cells in lead-treatment groups could be because of capacitation inhibition. In view of differential effects of lower and higher concentrations of lead on phosphorylation of tyrosine containing proteins in buck sperm cells, further studies are required especially on identification of the specific subset of proteins which are expressed more or less following exposure to lead so as to precisely unravel the effect of lead on specific marker proteins.

Sperm morphology assessment is essential for certification of semen quality for better fertilization results [91] and sperm morphology changes are known to impair fertility [92]. Therefore, to unravel whether lead produces any morphological or ultrastructural changes or defects in spermatozoa which in turn may be responsible for lead-induced alterations in viability and functional dynamics of buck spermatozoa, scanning and transmission electron microscopy studies were undertaken. Scanning electron micrographs revealed irregular plasma membrane at the head and middle piece of sperms after 15 min and acrosome appeared swollen and middle piece appeared wavy after 3. Compared to the effect of lead at 5 ppm after 15 min, morphological defects were far more severe after 3 h and characterized by swollen tail, bent neck, focal areas of membrane rupture on sperm head surface and deformed middle piece. Integrity of plasma membrane in the head region was disturbed and few sperm cells even showed swollen plasma membrane and tail in 20 ppm group. Compared to buck spermatozoa, sharp depression and granular texture on sperm cell surface has been reported in humans working in battery and paint factories, [34]. Similar alterations in morphological structure of sperm head and compromised development of embryos have recently been reported following exposure to mercury in fish [93]. Almost similar, but somewhat more marked alterations in buck spermatozoa structures have also been reported from our laboratory following in vitro exposure to mercury [3]. Thus, evidently implying lead -induced damage to plasma membrane of sperm head, mid piece and tail may be responsible for alterations in vitality and functional dynamics of sperm cells as sperm cells with damaged plasma membrane are not functionally viable.

Transmission electron microscopy (TEM) is another authentic and valuable tool to elucidate structural intactness and sub-cellular structures of cells. Lead inflicted concentration-dependent structural and substructural defects in sperm head membrane, acrosome and middle piece which further increased with increase in exposure time. Our findings of SEM and TEM studies are in agreement with almost similar damage to sperm head membranes, and acrosome breakage with formation of various sized macrovesicles following exposure to arsenic, cadmium, mercury and platinum [94]. Large round holes in arsenic, cadmium and chromium exposed groups, and numerous folds in acrosome membrane in vanadium-treated rabbit spermatozoa [95]. Deformities in middle piece region like distorted outer dense fiber, swollen mitochondria having collapsed mitochondrial cristae could be responsible for low mitochondrial potential and reduced physiological functions like progressive motility and alterations in certain kinematics patterns (VSL, VCL, VAP & BCF) of sperm cells. Similar disruption in mitochondrial organization, loss of cristae, electron-lucent matrix in mitochondria, vesicles of varied sizes over the cell surface and convoluted sperm cells of *A. crassispina* (sea urchin species) have been reported following 60 min exposure to 5–10 ppm cadmium [96].

Ca^2+^ signal transduction regulates different cellular functions in eukaryotic cells including transcription, enzyme activities, protein phosphorylation, cell death etc. [97]. Increase in intracellular cytosolic Ca^2+^ ([Ca^2+^]_i_) concentration is critical for different physiological processes in sperms such as motility and acrosome reaction which ultimately govern the fertilizing capacity of sperms [98]. Increase in [Ca^2+^]_i_ in cells may be due to its release from intracellular stores or influx through different Ca^2+^ channels across plasma membrane, mainly the voltage-operated calcium channels (VOCC; [99]) and store-operated channels [100]. Both these processes replenish the intracellular Ca^2+^ stores and mediate long-term cytosolic Ca^2+^ signals [101]. Major function of VOCCs is to convert the changes in membrane potential into intracellular signals involving Ca^2+^ and play important role in ion-influx into sperm [98]. Heavy metals like cadmium, mercury and lead are great threat for mammalian cells including sperm cells as these are able to replace and/or mimic the essential metals, especially calcium, during early steps of cellular functions especially transport and metabolism but are incapable of mediating subsequent vital steps in cellular functions [102, 103]. Inhibitory effect of lead on spermatozoa motility may possibly be attributed to its ability to rapidly pass through the voltage-operated calcium channels (VOCC) and allowing the VOCCs to remain open and result in hyperpolarization [40, 73, 104]; and thus, decrease in sperm cells motility. Almost similar inhibitory effects of mercury on motility and vitality of buck spermatozoa have been reported from our laboratory [3, 24].

## Conclusions

Lead decreased sperm motility, viability, and induced premature/spontaneous acrosome reaction by increasing intracellular Ca^2+^ release, and protein tyrosine phosphorylation in concentration-and time-dependent manner. It lowered progressive motility and altered kinematic patterns of sperm cells, damaged plasma membrane, acrosome, mid-piece, tail, and mitochondrial ultrastructures, and induced apoptosis. Lead seemingly interacts with L- and T- type Ca^2+^ channels and/or mimics Ca^2+^ to enter the cell through calcium channels and results in hyperpolarization and premature acrosome exocytosis of sperm cells. All these effects altogether reduce the capacitation competence of spermatozoa; and thus, adversely affect the fertilizing capacity of spermatozoa.

## Material and methods

Present study was conducted using freshly collected semen from six healthy adult *Barbari* bucks aging between two and four years and weighing from 25 to 35 kg. Animals were maintained under standard semi-intensive management system. Guidelines of the Committee for the Purpose of Control and Supervision of Experiments on Animals (CPCSEA) were followed as per approval of the Institutional Animal Ethics Committee of DUVASU (IAEC No. 110/IAEC/16 dated 16/09/2016) in compliance of the guidelines of CPCSEA.

1. *Semen collection and analysis*: Total ninety semen ejaculates, twice a week from each buck, were collected from experimental bucks using artificial vagina (AV) with graduated semen collection cup from March to May and September to November. Immediately after collection, semen samples were kept in CO_2_ incubator at 37 ℃, then processed over thermostatically regulated stage at 37 ℃ and examined under phase-contrast microscope. Semen ejaculates showing mass motility ≥ 3, sperm motility of ≥ 80% and abnormal morphology of ≤ 10% were used for further detailed investigation as established in our laboratory [3, 24].
2. *Semen dilution*: After initial evaluation of the apparent quality of semen based on viability and motility of spermatozoa, semen was diluted using PBS having 0.5% glucose (Ca^2+^ and Mg^2+^ free, pH-7.4) [3] to have the final concentration of 40 × 10^6^ spermatozoa/ml. The diluted semen was divided into experimental groups (40 × 10^6^ spermatozoa/ml) for further studies.
3. *Experimental design*: Based on the information available in literature on lead concentrations (0.2 to 2.65 ppm) in semen of different species of animals including human beings [105–107], eight different concentrations (0.25 ppm, 0.5 ppm, 1.0 ppm, 2.5 ppm, 5.0 ppm, 10 ppm, 20 ppm and 50 ppm) of Pb were selected for pilot study (Supplementary data). We selected four different concentrations of lead acetate (0.5 ppm, 5.0 ppm, 10 ppm, 20 ppm) based on live percent and progressive motility of spermatozoa to study the effect of lead acetate on different functional parameters of goat spermatozoa after in vitro exposure for 15 min and 3 h. Stock solution (1 mg/ml) of lead acetate [Pb (CH_3_COO)_2_ 3H_2_O, having 54.56% lead], was prepared in normal saline (NS).
4. *Effect on viable count of spermatozoa*: To ascertain the effect of lead on percentage of viable spermatozoa following exposure to lead acetate at 0.5, 5, 10, and 20 ppm for 15 min, 1 h and 3 h, Eosin-Nigrosin staining technique was employed as per the method described earlier by Kushawaha et al. [24]. At least 200 spermatozoa were counted and identified as live or dead under oil-immersion (100X) objective. Pink stained (eosinophilic) spermatozoa against the dark background were considered as dead and those which remained unstained against dark background of nigrosine were counted as live and the percentage of live spermatozoa was calculated using the following equations:
  $$\% {\text{ viable spermatozoa }} = \frac{{{\text{Total no}}.{\text{ of viable spermatozoa}}}}{{{\text{Total no}}.{\text{ of spermatozoa observed}}}} \times { 1}00$$
5. *Effect on progressive motility and motion kinematic patterns of spermatozoa*: Motility and motion kinematic characteristics of spermatozoa in semen samples at different treatment groups of lead acetate were evaluated using Computer Assisted Semen Analyser (CASA) up to 3 h at 37 ℃ in dry bath as previously describe by our lab [3]. Briefly, 5 μl of diluted semen sample was loaded in the metallic sperm counting chamber with surface graticule of 100 × 0.01 sq mm (sperm processor, Welcomenagar, India) and 6 fields was acquired for assessment of the motility for each aliquot. Rapid progressive motility (%), and motion kinematic patterns of spermatozoa like curvilinear velocity (VCL; µm s^−1^), straight-line velocity (VSL; µm s^−1^), average path velocity (VAP; µm ^s−1^), linearity (LIN; %), straightness (STR; %), beat cross frequency (BCF; Hz), wobble (WOB; %), and maximum amplitude of head lateral displacement (maxALH; µm), compared to the control group. was determined using CASA (Biovis-2000, Version V 4.59, developed by Expert Vision Labs. Pvt. Ltd., Mumbai, India, URL: http://www.expertvisionlabs.com/BiovisCASA.html), sperm counting chamber, negative phase contrast and 10X objective. Settings of CASA system were fixed using algorithm based on size, shape, and detection of goat buck sperm head as follow: X (pixels/unit)- 1.905 µ, Y—(pixels/unit) 1.905 µ, Camera frequency (FPS)-160, Frame rate- 60, Frames acquired- 60, Non progressive limit- 0–10(µm/s), Slow progressive limit- 10–25(µm/s), Rapid progressive limit- > 25 (µm/s), Maximum velocity for tracking- 150 (µm/s), Minimum VCL- > 25(µm/s), Minimum VAP- > 10 (µm/sec), Minimum VSL > 1(µm/s), Minimum track Length—51(% of frames), Area 1–9999 µm, Aspects 0–99999 µm, Axis major 5–16 µm, Axis minor 3–10 µm, Compactness 0–50, Size of image-1280 × 960 pixels. A minimum of 300 sperm cells and six fields were analysed for each aliquot.
6. *Effect on acrosomal integrity*: Acrosomal integrity of sperm cells was evaluated by employing the FITC-PSA staining method of Mendoza et al. [108]. Briefly, diluted semen samples (40 × 10^6^/ml) were suspended in PBS (Ca^2+^ and Mg^2+^ free) and centrifuged at 220 × g for 3 min. Sperm’s pellet was washed three times in PBS (pH = 7.4). After three washings, the sperm pellet was resuspended in PBS. 10 μl of the sample was taken and a thin and uniform smear was prepared on clean grease-free glass slide. The slide was kept for air drying. Air dried slides were incubated with 100 μg/ml FITC-PSA (Sigma) in PBS (pH = 7.4) for 30 min at room temperature in dark. Slides were removed and air dried. After air drying, slides were observed using Nikon Eclipse TE 2000-S microscope with phase contrast and epifluorescence optics under FITC/blue filter. Two hundred spermatozoa per slide from 3 replicates were differentiated according to the fluorescence patterns in their acrosomes (bright fluorescence acrosome-intact, no fluorescence or only fluorescence of the equatorial segment means loss of acrosome).
7. *Effect on capacitation and acrosome-reaction status*: Capacitation and acrosome reaction status in spermatozoa were assessed using chlortetracycline (CTC) staining [109]. Chlortetracycline-labeled spermatozoa suspension was placed on a glass slide for examination using Nikon Eclipse TE 2000-S microscope with phase contrast and epifluorescence optics under blue-violet illumination (excitation at 400–440 nm and emission at 470 nm by using 40 × objectives). A total of 200 spermatozoa per slide from 3 replicates were observed. On examination under microscope, three different forms of CTC pattern were observed:
8. *Pattern F*: Even distribution of fluorescence over the entire head indicated non-capacitated spermatozoa with intact acrosome.
9. *Pattern B*: Fluorescence-free band in the post acrosomal region and fluorescence in anterior portion of the head indicated capacitated spermatozoa having intact acrosome.
10. *Pattern AR*: Acrosomal region did not fluoresce, whereas the post-acrosomal segment either showed fluorescence or no fluorescence which indicated capacitated and acrosome-reacted spermatozoa.
11. *Effect on Mitochondria Transmembrane Potential (MTP)*: Mitochondrial transmembrane potential in sperm cells was evaluated by using the Mito Probe JC-1 Assay Kit (M 34152, Invitrogen, ThermoFischer Scientific). The standard protocol specified by the manufacturer was followed for evaluation of the MTP in sperms. Highly sensitive flurochorme JC-I was used for detection of mitochondrial transmembrane potential. With higher inner transmembrane potential, JC-I accumulates inside the mitochondria and emits orange red fluorescence, whereas the low transmembrane mitochondrial potential causes JC-I to get its monomer formed and fluoresces green. To prepare the reagent JC-1 powder and DMSO solutions were allowed to come to room temperature before use. 200 μM JC-1 stock solution was prepared immediately prior to use by dissolving the contents of one vial in 230 μl of the DMSO provided. For each sample, sperm cells pellet was suspended in 1.0 ml phosphate-buffered saline to have the strength of approximately 1 × 10^6^ cells/ml. 10 μl of 200 μM JC-1 (2 μM final concentration) was added and incubated the cells at 37^0^C in 5% CO_2_ incubator for 15 to 30 min. Slides were observed using Nikon Eclipse TE 2000-S microscope with phase contrast and epifluorescence optics under blue-violet illumination (excitation at 400–440 nm and emission at 470 nm by using 40 × objective). A total of 200 sperm cells per slide were observed from 3 individual replicated.
12. *Effect on DNA Fragmentation*: The APO-BrdU™ TUNEL Assay was used to detect the DNA fragmentation as per manufacturer’s instructions. The APO-BrdU TUNEL Assay Kit (A23210, Invitrogen) was used to detect mercury-induced DNA fragmentation in spermatozoa following in- vitro exposure to lead acetate (0.5, 5, 10, and 20 ppm) for 15 min and 3 h. After treatment, semen samples were centrifuged at 1500 rpm for 5 min in DPBS (twice) to remove free lead from the media by removing the supernatant. DNA fragmentation was assessed as per manufacturer’s instructions. Briefly, 5 ml 1% (w/v) paraformaldehyde in PBS was added to 1–2 × 10^6^ sperm cells (in 0.5 ml of phosphate-buffered saline) and placed on ice for 15 min. Samples were centrifuged for 5 min at 300×*g* and the supernatant were discarded. Cells were washed in 5 ml PBS, and then centrifuged to make the pellet and this step was once again repeated to obtain the pellet of treated cells. Cells were re-suspended in 0.5 ml of PBS. To this, 5 ml of ice-cold 70% (v/v) ethanol was added, and the cells were allowed to stand for a minimum period of 30 min in − 20 °C freezer. Total 50 µL DNA-labelling solution was prepared for each sample by adding 10 µL of the reaction buffer, 0.75 µL of TdT enzyme, 8.0 µL of BrdUTP and 31.25 µL of distilled water in tube and mixed well. Re-suspended the cell pellets of each tube in 50 µL of the DNA-labelling solution. The DNA-labelling reaction was carried out at 22–24 °C overnight. After incubation, 1.0 ml rinse buffer was added to each tube and centrifuged at 300×*g* for 5 min. Supernatant was removed by aspiration and this step was repeated by rinsing the cells with 1.0 ml of rinse buffer. Centrifuged the samples at 300×*g* and removed the supernatant by aspiration. 100 µL of the antibody staining solution for each sample was prepared by mixing 5.0 µL of the Alexa Fluor 488 dye labelled anti-BrdU antibody with 95 µL of the rinse buffer. Cells pellet was re-suspended in 100 µL of the antibody solution and kept at room temperature for 30 min, and during incubation samples were protected from light. 0.5 ml of propidium iodide, a staining buffer, was added to each sample and the cells were incubated again at room temperature for an additional period of 30 min while protecting from light exposure. Slides were observed using Nikon Eclipse TE 2000-S microscope with phase contrast and epifluorescence optics under blue-violet illumination (excitation at 400–440 nm and emission at 470 nm by using 40X objective). A total of 200 sperm cells per slide were observed from 3 individual replicated.
13. *Effect on cholesterol levels (mg/ml)*: Control and lead acetate (0.5–20 ppm) treated semen samples were processed for measuring cholesterol level as per method of Folch et al. [110]. Briefly, control and lead-treated semen samples (50 million sperm cells in each group) were centrifuged and washed twice with 0.5 ml PBS at 5000 rpm for 3 min to remove lead from the media and then contents of each tube were transferred to 15 ml tube. To this, 0.9 ml chloroform: methanol (2:1, v/v) containing 0.20 ml acetic acid/100 ml) was added. Then the tubes were shaken mechanically for 20 min. Thereafter 2.0 ml distilled water was added and again shaken for 10 min. Centrifugation was done for 15 min at 4000×*g*, and the supernatant was discarded. After that, 5.0 ml chloroform: methanol: distilled water (3:48:47, v/v/v) was added to each tube and shaken for 10 min; then centrifuged the tubes for 15 min at 4000×*g*. Supernatant was discarded and infratnt was filtered through glass pipette containing plug of glass-wool to remove any residual proteins. Glass wool was then rinsed with 1 ml chloroform and the sample was dried at room temperature. Then the sample was reconstituted in 0.5 ml chloroform and estimation of cholesterol was performed by colorimetric method as per the procedure described in kit (Autospan Liquid Gold cholesterol). Three replicates per sample were used. Chloroform was used as a blank. Absorbance was measured at 510 nm and calculated as total cholesterol concentration (mg/dl) = Absorbance of Test/Absorbance of Standard × 200 by using Semi-automatic Biochemistry Analyzer Chem-7 (Erba, Mannheim, Germany).
14. *Effect of lead acetate on apoptosis*: Image-iT™ LIVE Green Caspase-3 and -7 Detection Kit (I 35106, Invitrogen) was used to detect caspase 3 and 7 positive cells. A distinctive feature of the early stages of apoptosis is activation of caspase enzymes, the name applied to cysteine-aspartic acid specific proteases (Caspase). These enzymes participate in series of reactions that are triggered in response to pro-apoptotic signals and result in cleavage of protein substrates and in subsequent disassembly of the cell. The recognition sequence in the target substrate always includes an aspartic acid residue; cleavage takes place at the carbonyl end of that residue. The Image-iT™ LIVE Green Caspase-3 and -7 Detection Kit employ a novel approach to detect active caspases that is based on fluorescent inhibitor of caspases (FLICA™) methodology, essentially an affinity label. The reagent associates a fluoromethyl ketone (FMK) moiety, which can react covalently with cysteine, with a caspase-specific amino acid sequence. This recognition sequence is aspartic acid-glutamic acid-valine-aspartic acid (DEVD) for the caspase-3 and-7 reagent. A carboxyfluorescein group is attached as a reporter. The FLICA reagent is thought to interact with the enzymatic reactive centre of an activated caspase via recognition sequence, and then to attach covalently through the FMK moiety. Unbound FLICA molecules diffuse out of the cell and are washed away; the remaining green, fluorescent signal is a direct measure of the amount of active caspase that was present at the time the inhibitor was added. Briefly, 250 µL of the diluted semen samples at a concentration of 40 × 10^6^/ml were washed three times in PBS (pH = 7.4). After three washings, the sperms pellet was re-suspended in PBS. 30-fold dilution of 30X FLICA reagent working solution was added to cell suspension to cover the cells. Cells were incubated for 60 min under existing culture conditions and protected from light. Thereafter, the solution was removed, and the cells were gently rinsed with PBS. Then cells were washed twice with 1X wash buffer. 200 cells/slide from 3 replicates were observed under a fluorescence microscope using EVOS-FLoid imaging system, an inverted epi-fluorescence digital microscope (Invitrogen, USA) with GFP filter (excitation 470/22 nm and emission at 510/42) at 20 × magnification.
15. *Effect of lead acetate on cAMP levels*: Enzyme Immunoassay (EIA) Direct cyclic AMP kit (CA200-Sigma) acetylated version was used to determine adenosine 3, 5-cyclic monophosphate (cyclic AMP; cAMP) in sperm cells. Cyclic-AMP is one of the most important “second messengers” which is involved as modulator in cellular physiological processes. Effect of lead acetate on cyclic AMP levels in spermatozoa was assessed using secondary antibody coated multi well ELISA plate employing the assay procedure as per the instructions provided in the kit (CA201, Sigma St. Louis, USA). The measured optical density was used to calculate the concentration of cAMP in samples as per the method given in assay-kit.
16. *Effect of lead acetate on intracellular calcium (*_*i*_*Ca*^*++*^*) levels*: For measurement of the intracellular calcium levels**,** Fura-2 AM (F0888, Sigma) by using flow cytometer (FACS Diva, Version-7. 0.1.BD-Bioscience, USA) as described earlier [24]. Permeabilization medium (0.5% Triton-X and 0.02% Proteinase K in cold methanol) was used for permeabilization of Fura-2 AM in sperm cells, following which the sperm cells were fixed in the fixative solution (1.76% paraformaldehyde). Briefly, 1 mM Fura 2-AM stock solution was prepared by dissolving the contents of vial in acetone. A total of 8 μl of Fura 2-AM solution was added to sperm suspension containing 250 μl of Triton-X and 250 μl of PBS (10 μM Fura 2-AM final concentration) and the sperm/Fura 2-AM suspension was kept at room temperature for 2 h in dark to permit loading of Fura 2-AM into the sperm. Before flow cytometer analysis, cells were washed with PBS at 2000 rpm for 10 min and then the pellet was resuspended in 500 μl of sheath buffer in FACS tubes. Gating and exclusion of false positives were performed by using unstained samples and 10,000–15,000 events of cells were used to determine the intracellular level of calcium based on forward versus right angle scatter. Fura 2-AM emission was detected at 495 nm/519 nm by using FL1 filter.
17. *Effect of lead acetate on tyrosine-phosphorylated proteins*: Sperm lysates of the control and different lead-treated groups were evaluated for detection of tyrosine-phosphorylated proteins using immuno-blotting as reported earlier from our laboratory [111]. Briefly, total protein (20 μg) was extracted from different sperm lysates by using the commercial total cell lysis protein isolation buffer (Amresco, USA). The electrophoresis (10% gel) was carried out at room temperature by using the constant current and 70 V of voltage for 3.0 h. The membrane was transferred to the blocking solution (2.5% of skim-milk powder prepared in Tris-buffered saline with Tween-20 and sodium orthovanadate, TBS-TV: 20 mM Tris, 150 mM NaCl, pH 7.6; 0.1% (v/v) Tween-20, 1 mM Na_3_VO_4_, TBST) and kept overnight with slow agitation at 4 °C. After overnight blocking, the membrane was removed from the blocking solution and then washed slowly in the TBST. The membrane was treated with primary antibody (Monoclonal Anti-Phosphotyrosine, P5872, clone PT-66, Sigma Aldrich) for the corresponding tyrosine phosphorylation. Primary antibody dilution (1:500) was carried out in TBST. Treatment with the primary antibody was carried out for overnight at 4 °C. The washed membrane was treated with the secondary antibody (Goat anti-mouse IgG HRP, A4416, Sigma, 1:5000 in TBST) for 2 h at 37 °C. Development of different protein bands was carried out by using DAB substrate (SIGMA-FAST DAB tablets, St Louis, USA) as per the instructions written on the packing. Relative molecular weights of different protein bands were determined using broad-range molecular weight markers (pre-stained protein molecular weight, New England Biolabs) and gel documentation and analysis system.
18. *Effect on immuno-localization of TP proteins*: Immunolocalization of tyrosine phosphorylated proteins was carried out by employing indirect immune fluorescence technique as described by Swain et al. [111]. Briefly, after treatment sperm cells were washed with PBS and were smeared on glass slides and allowed to air dry. The air-dried slides were fixed in 4% paraformaldehyde in phosphate buffered saline (PBS, pH 7.4) for 30 min and followed by rinsing with PBS. The rinsed slides were permeabilised with cold methanol for 30 min at 4 °C. After permeabilization, slides were again rinsed with PBS. Nonspecific sites on the slides were blocked using 5% BSA in PBS for 2 h at room temperature followed by rinsing with PBS. The smeared slides were incubated with mouse monoclonal anti-phosphotyrosine antibody (Monoclonal Anti-Phosphotyrosine P5872, Sigma; Clone PT-66, diluted (1:50) in 1% BSA in PBS) for 4 h. After incubation with primary antibody, the slides were rinsed with PBS and incubated with secondary antibody (anti mouse IgG- FITC conjugate, Sigma; F4018; diluted (1:5000) in 1% BSA in PBS for 2 h at 37 °C in dark. Following two hours of incubation, slides were rinsed with PBS three times and then processed for observation under microscope. Cover slip was mounted on the smeared slide with antifading medium (1.5% w/v DABCO, Sigma Aldrich, St Louis, MO, USA in 90% v/v glycerol). Slides were observed using EVOS-FLoid imaging system, an inverted epi-fluorescence digital microscope (Invitrogen, USA) with GFP filter (excitation 470/22 nm and emission at 510/42) at 20 × magnification A total of 200 sperm cells per slide from 3 replicated were observed and different localization patterns of spermatozoa were evaluated as per the sites of fluorescence on sperm cells [24].
19. *Effect of lead acetate on apoptosis at gene expression levels*: Semen samples (40 × 10^6^ spermatozoa/ml) of control and lead-treatment groups were centrifuged at 2000 rpm for 10 min to remove free lead from the media and obtain the pellet of spermatozoa. Lysis of the somatic cells was done by using 1 ml of 0.5% Triton X-100 to the pelleted spermatozoa and tubes were briefly vortexed. Then these were incubated at 37 ℃ for 10 min in an incubator. Following incubation, the spermatozoa were again pelleted by centrifugation at 2000 rpm for 10 min. RNA extraction was carried out by Gene JET RNA Purification kit (Gene JET K0731, Thermoscientific) as per manufacturer’s instructions for complete dissociation of cell membrane. Depending on the size of pellet, RNA was re-dissolved in 15–30 μl of nuclease-free water (Amresco, USA). RNA samples were stored at − 80 °C for further analysis. The cDNA was synthesized in thermal cycler (Bio-Rad) from the isolated RNA samples using Revert Aid First Strand cDNA Synthesis Kit (K1622, ThermoFischer Scientific, USA) following manufacturer’s instructions.
20. *Primer sequences*: To amplify the desired genes, specific primers were selected from the already published primer sequences [112]. These primers were further aligned by using PRIMER BLAST at NCBI; details of which have been given below (Table 4).
21. *Real-time PCR*: Quantitative Real-time PCR (CFX96 Touch Real-Time PCR Detection System, Bio-Rad, USA) was used for gene-expression studies. For each gene, samples were run in duplicate. The reactions were performed with 100 ng of cDNA, 5 μl of PowerUp™ SYBR™ Green Master Mix, 1 μl of each primer (10 pmol) in 10 μl of final volume. Denaturation was performed at 95 °C for 1 min, annealing at 61 °C for Bcl-2, 60 °C for Bax and β-actin for 1 min and final extension at 72 °C for 30 s with 40 cycles. The relative expression of PCR product was determined by the equation 2^−ΔΔCt^ method [113].
22. *Electron microscopy (SEM & TEM)*: Electron microscopy of the semen samples of control and lead-treatment groups was performed employing the method described earlier [3]. Briefly, after exposing the semen samples to different concentrations of lead for pre-defined periods (15 min and 3 h), semen samples were centrifuged twice at 1500 rpm for 5 min in DPBS to remove lead from the medium. Then pellets were resuspended in the mixture of 2% paraformaldehyde and 2.5% glutaraldehyde in 0.1 M phosphate buffer (pH 7.4) for 12 h at 4 ℃ for fixation. After that samples were centrifuged at 1500 rpm for 10 min and the supernatant was discarded. The pellet was resuspended in DPBS, centrifuged, and washed. Within 2 h, the samples were taken to All-India Institute of Medical Sciences (AIIMS) for imaging. The pellet was suspended in buffer, centrifuged, and washed. It was re-suspended in buffer and a drop of it was spread on a cover slip. The samples were air-dried, sputter coated (SCD 050 Super Cool Sputter System; Baltec Technology, Liechtenstein) with colloidal gold and observed under an EVO 18 Zeiss scanning electron microscope at an operating voltage of 20 kV. Images were digitally acquired by using the Smart SEM software. Tin sections of grey-silver colour interference (70–80 nm thick) were cut and mounted onto 300 mesh copper. The sections were stained with uranyl acetate and alkaline lead citrate, then gently washed with distilled water, and observed at Sophisticated Analytical Instrumentation Facility (SAIF), All India Institute of Medical Sciences, New Delhi under Tecnai G2 20 S-Twin transmission electron microscope (Fei Company, The Netherlands) at an operating voltage of 200 kV. Images were digitally acquired by using camera and Digital Micrograph software attached to the microscope.
23. *Effect of lead acetate on Ca*^*2+*^* channels*
24. *Effect of Nifedipine (L-type Ca*^*2+*^* channel blocker) alone and in the presence of lead acetate*: Stock solution of nifedipine (10^–1^ M) was prepared in DMSO. Effect of nifedipine was studied as per the following experimental protocol: Group 1—Control, Group 2—Vehicle Control (0.5% DMSO), Group 3—Nifedipine alone (500 µM), Group 4—0.5 ppm lead acetate ± 500 µM Nifedipine, Group 5—5 ppm lead acetate ± 500 µM Nifedipine, Group 6—10 ppm lead acetate ± 500 µM Nifedipine, Group 7—20 ppm lead acetate ± 500 µM Nifedipine. Sperm cells were pre incubated for 15 min with Nifedipine then with different concentrations of lead acetate as per the details given above groups. Semen samples of all the groups were examined for motility employing the standard methods of CASA for 15 min and 3 h.
25. *Effect of NNC 55-0396 hydrate (T-type Ca*^*2+*^* channel blocker) alone and in the presence of lead acetate*: Stock solution (10^−2^ M) of NNC was prepared in ultrapure water. Working solution (250 × 10^–6^ M) was prepared in PBS. Effects of NNC alone and in the presence of lead acetate were studied employing the following experimental protocol: Group 1—Control, Group 2—NNC alone (10 µM), Group 3—0.5 ppm lead acetate ± 10 µM NNC, Group 4—5 ppm lead acetate ± 10 µM NNC, Group 5—10 ppm lead acetate ± 10 µM NNC, Group 6—20 ppm lead acetate ± 10 µM NNC. Sperm cells were pre incubated for 15 min with NNC then with different concentrations of lead acetate as per the details given above groups. Semen samples of all the groups were examined for motility employing the standard methods of CASA for 15 min and 3 h.

**Table 4:** Primer sequences used for qRTPCR for gene expression

Gene	Sequence of Primers	EMBL/reference	Product length (bp)	Annealing temp
Bcl-2-F	5′-TGCTGCTGTTTCTGCCTACA-3′	NM_001166486.1	143	61^ͦ^ C
Bcl-2-R	5′-GCACTTTTGCATGGGTCAA-3′			
Bax-F	5′-CATGGAGCTGCAGAGGATGA-3′	XM_002701934.1	100	60 ^ͦ^ C
Bax-R	5′-GTTGAAGTTGCCGTCGGAAA-3′			
β-actin F	5′-AGTTCGCCATGGATGATGA-3′	Dangi et al. [112]	54	60 ͦ C
β-actin R	5′-TGCCGGAGCCGTTGT-3′			

## Statistical analysis

Statistical analysis of data was performed using SPSS Version 19.0 and applying one-way *ANOVA* and Tukey’s test, where  p < 0.05 was considered significant. The analysis of relative mRNA expression data across different groups was based on crossing point (Cp) values. The Cp values of each gene were subtracted from the arithmetic mean value of Cp of β-actin to calculate ΔCt. Results have been expressed as mean ±SE (p < 0.01). Relative expression of PCR product was determined by the equation 2^−ΔΔCt^ [113].

## Data Availability

Data will be available upon request. Further information and requests for resources and reagents should be directed to the Dr Rajkumar Singh Yadav rajan.vaday@gmail.com.
